# CSYseq: The first Y-chromosome sequencing tool typing a large number of Y-SNPs and Y-STRs to unravel worldwide human population genetics

**DOI:** 10.1371/journal.pgen.1009758

**Published:** 2021-09-07

**Authors:** Sofie Claerhout, Paulien Verstraete, Liesbeth Warnez, Simon Vanpaemel, Maarten Larmuseau, Ronny Decorte

**Affiliations:** 1 Forensic Biomedical Sciences, Department of Imaging & Pathology, KU Leuven, Leuven, Belgium; 2 KU Leuven, Department of Mechanical Engineering, Noise and Vibration Engineering, Leuven, Belgium; 3 DMMS Lab, Flanders Make, Heverlee, Belgium; 4 Histories vzw, Mechelen, Belgium; 5 Department of Human Genetics, KU Leuven, Leuven, Belgium; 6 Laboratory of Forensic genetics and Molecular Archaeology, UZ Leuven, Leuven, Belgium; National Institute of Genetics, JAPAN

## Abstract

Male-specific Y-chromosome (chrY) polymorphisms are interesting components of the DNA for population genetics. While single nucleotide polymorphisms (Y-SNPs) indicate distant evolutionary ancestry, short tandem repeats (Y-STRs) are able to identify close familial kinships. Detailed chrY analysis provides thus both biogeographical background information as paternal lineage identification. The rapid advancement of high-throughput massive parallel sequencing (MPS) technology in the past decade has revolutionized genetic research. Using MPS, single-base information of both Y-SNPs as Y-STRs can be analyzed in a single assay typing multiple samples at once. In this study, we present the first extensive chrY-specific targeted resequencing panel, the ‘CSYseq’, which simultaneously identifies slow mutating Y-SNPs as evolution markers and rapid mutating Y-STRs as patrilineage markers. The panel was validated by paired-end sequencing of 130 males, distributed over 65 deep-rooted pedigrees covering 1,279 generations. The CSYseq successfully targets 15,611 Y-SNPs including 9,014 phylogenetic informative Y-SNPs to identify 1,443 human evolutionary Y-subhaplogroup lineages worldwide. In addition, the CSYseq properly targets 202 Y-STRs, including 81 slow, 68 moderate, 27 fast and 26 rapid mutating Y-STRs to individualize close paternal relatives. The targeted chrY markers cover a high average number of reads (Y-SNP = 717, Y-STR = 150), easy interpretation, powerful discrimination capacity and chrY specificity. The CSYseq is interesting for research on different time scales: to identify evolutionary ancestry, to find distant family and to discriminate closely related males. Therefore, this panel serves as a unique tool valuable for a wide range of genetic-genealogical applications in interdisciplinary research within evolutionary, population, molecular, medical and forensic genetics.

## Author summary

Around 95% of the male-specific Y-chromosome (chrY) is non-recombining and therefore inherited in a conserved manner from father to son. It can therefore serve as a powerful marker for interdisciplinary genetic-genealogical research as it provides a strong link between genetic information and a family tree or pedigree. While Y-chromosomal short tandem repeats (Y-STRs) discriminate close paternal kinships, single nucleotide polymorphisms (Y-SNPs) enables the identification of far evolutionary ancestry. Unfortunately, an extensive chrY-specific sequencing panel combining a large number of familial Y-STRs and evolutionary Y-SNPs was not yet available. Therefore, chrY is rarely included in research projects and not often linked to a genealogical, history-demographical or life science database. In this way, the importance of chrY still remains not yet fully understood. Massive parallel sequencing (MPS) allows the simultaneous analysis at sequence level of Y-SNPs and Y-STRs with variable mutation rates in a large number of males. However, up until today, no commercial kit is exploiting the full potential that MPS offers on chrY. Therefore, we developed the ‘CSYseq’, which is the first extensive chrY-specific sequencing panel. The CSYseq simultaneously identifies 9,014 slow mutating Y-SNPs to identify evolutionary ancestry, and 202 rapid mutating Y-STRs to investigate paternal relationships. We validated and optimized the panel through the analysis of 130 males distributed over 65 families. This novel MPS panel is useful for biogeographical identity and ancestry analysis, together with Y-chromosome profiling for the identification of patrilineages and discrimination of closely related males. As the CSYseq includes a very diverse set of markers that can be easily interpreted, it is interesting for different interdisciplinary applications within evolutionary, population, molecular, medical and forensic genetics.

## Introduction

For a long time, male-specific Y-chromosome (chrY) polymorphisms have been widely investigated for their distant and close paternal lineage identification in various fields such as anthropology, evolutionary biology, population genetics, genetic-genealogy and forensic sciences [1–4]. As 95% of chrY does not recombine with chrX (NRY), it is inherited from father to son in a conserved manner. However, passing on the Y-chromosome over generations allows DNA variation to be accumulated during spermatogenesis. Genetic chrY variation on the NRY is caused by DNA modifications, such as replication slippage or base pair (bp) substitutions. Commonly typed chrY modifications are single nucleotide polymorphisms (Y-SNPs) and short tandem repeats (Y-STRs) [5,6].

Y-SNPs are slowly mutating bi-allelic markers (on average 10^−8^ to 10^−9^ mutations per generation, mpg) with a single-base variation useful for predicting human ancestry and origins as well as studying evolutionary migration patterns [5,7–9]. They enable the reconstruction of a well-preserved male phylogenetic tree divided into 20 main Y-haplogroups (from ‘A’ to ‘T’) and currently more than 9,000 Y-subhaplogroups [10]. Some Y-SNPs were identified more recently, which means that they can be attributed to a specific population or even a single family [6]. In 2014, Scozzari *et al*. sequenced approximately 1.5 Mb of the NRY using 68 unrelated males covering all major Y-haplogroups. They discovered eight private substitutions causing amino acid changes in protein-coding genes and approximately 1,900 novel Y-SNPs [11]. To date, more than 700,000 Y-SNPs have been detected according to the ISOGG YBrowse database (International Society of Genetic Genealogy human Y-chromosome Browser, ybrowse.org/gb2/gbrowse/chrY), and high-throughput analyzing techniques such as next generation sequencing (NGS) ensure that this number is continuously increasing. Due to the growing Y-SNP discovery rate, the entire phylogenetic tree becomes more complex. Therefore, Van Oven *et al*. constructed in 2014 a minimal version of the Y-tree which includes 417 branch-defining Y-SNPs. These Y-SNPs define the key phylogenetic positions and human evolutionary lineages around the world [10].

The other commonly typed DNA markers are the Y-STRs, which are fast mutating (10^−4^ to 10^−2^ mpg) multi-allelic variations. The high degree of variability is caused by DNA strand slippage during replication leading to an increase or decrease of the number of tandem repeats [12,13]. As a difference in one locus is sufficient to distinguish two close relatives, it is interesting to genotype multiple rapidly mutating (RM) Y-STRs [14–17]. In 2010, Ballantyne *et al*. identified 13 RM Y-STRs with a 6.5-fold higher mutation rate. These RM Y-STRs individualize more than 99% of 12,272 unrelated males from 111 worldwide populations and introduce a higher degree of haplotype diversity on a global scale [14]. Among these, there are multi-copy Y-STRs located in the palindromic regions of chrY [16,18]. Since mutation probability is higher across these Y-STRs [19], the level of discrimination between close paternally related individuals can be enhanced.

Analyzing both Y-SNPs and Y-STRs is interesting for interdisciplinary genetic-genealogical research and human population genetics. An example of its purpose in investigative genetic-genealogy is the pioneer solved cold case of Marianne Vaatstra in The Netherlands [2]. In this case, slow mutating Y-SNPs were genotyped in order to identify the Y-subhaplogroup and biogeographical origin of the perpetrator. This indicated that the murderer of Marianne was not an Asylum seeker, as was assumed in the village, but someone from the local area. Second, faster mutating Y-STRs were used later to find relatives of the perpetrator through a mass screening of male volunteers from the neighborhood. Genotyping slow mutating Y-STRs increased the chance of success to find a relative, but on the other hand, including rapidly mutating Y-STRs increased the discrimination power to distinguish two close relatives. For interdisciplinary genetic-genealogical research, including Y-SNPs is interesting because they could be important indicators for kinships (private and genealogical Y-SNPs), biogeographical origins and complex human traits [20]. The latter has already been confirmed in literature for complex human traits such as infertility, immune responses, cardiovascular risk and even COVID-19 mortality [21,22]. Complementary, Y-STRs are valuable to decrease false positive kinships, to confirm close biological family, to study their recent common ancestor relatedness and to differentiate between related and non-related males [23]. In human population and evolutionary genetics, the combination of Y-SNPs and Y-STRs enabled to analyze haplogroup-specific Y-STR mutation rates [24], recent and past migration events [25], biogeographical genetic variation [26], extra-pair paternity [27], network analysis within populations [28] and even correlations with socio-cultural factors [29].

Until now, chrY genotyping was mainly based on fragment analysis for Y-STRs or a single-base extension (SBE) assay for Y-SNPs using capillary electrophoresis (CE). But, CE has its limitations that can theoretically be overcome by high-throughput massive parallel sequencing (MPS) technology. First, Y-STRs of similar allele size but with a different sequence, called isoalleles, cannot be distinguished with CE. This results in unreported genetic variation between individuals or hidden parallel Y-STR mutations (PM) within genealogical pairs [30]. As MPS offers the ability to target and analyze DNA at sequence level, isoalleles can be distinguished, intra-repeat SNPs can be detected and new unique allelic variants of known STRs can easily be identified [31]. Second, due to spatial and spectral CE resolution, only a limited number of markers can be analyzed simultaneously resulting in the need to develop different multiplexes [2,32]. Currently, the two most comprehensive commercial CE-kits for Y-STR DNA profiling are the PowerPlex Y23 (23 Y-STRs, Promega) and the Yfiler Plus PCR Amplification Kit (27 Y-STRs, Applied Biosystems) [33]. With MPS, a large number of markers (both Y-SNPs and Y-STRs) can be analyzed simultaneously, reaching a higher discrimination capacity and wider range of applications [32,34]. To date, several MPS panels are already commercialized for SNP identity and ancestry analysis as well as STR marker DNA profiling.

Thermo Fisher Scientific was the first company to develop commercial kits for second-generation sequencing, with the Ion Torrent HID STR 10-plex being the first kit for autosomal STR genotyping [35]. In 2015, the kit was upgraded to the Early Access STR Kit v1, which was able to detect 25 autosomal STR loci [36]. Both kits are compatible with their Ion PGM platform. Also in 2015, Illumina developed the first targeted NGS panel, called the ForenSeq DNA Signature Prep kit, which targets alongside 58 STRs (including 27 autosomal STRs, 24 Y-STRs and 7 X-STRs) also 172 autosomal SNPs (94 identity SNPs, 56 ancestry SNPs and 22 phenotypic SNPs) [37]. For this kit, Illumina developed the MiSeq FGx System, which includes data analysis software [38] that provides investigators with additional genetic variation information [39]. Shortly after, Thermo Fisher Scientific developed the HID-Ion AmpliSeq Identity Panel to target 124 different SNPs (including 90 autosomal SNPs and 34 Y-SNPs), but no STRs. With this panel, the biogeographical ancestry can be determined through chrY analysis using the HID-Ion PGM system [40]. More recently, in 2019, Thermo Fisher Scientific commercialized the Ion AmpliSeq HID Y-SNP Research Panel v1, targeting 859 phylogenetic Y-SNPs where 640 Y-haplogroups can be determined [41]. The latter kit contains the largest number of Y-SNPs so far, but there is still no MPS panel that targets both evolutionary Y-SNPs as familial Y-STRs.

MPS offers the combination of sequencing large numbers of samples and markers while providing single-base sequencing information. The present study focusses on the development of the first extensive chrY-specific MPS panel, called the ‘CSYseq’. This newly developed panel targets a large number of phylogenetic informative Y-SNPs and multiple Y-STRs in a single assay. All Y-polymorphisms included in the panel were analyzed and investigated on their ease of interpretation, depth of coverage, discrimination power, mutability and chrY specificity.

## Results

To create our chrY-specific MPS panel, regions of interest containing known Y-STRs and reported Y-SNPs were carefully chosen based on literature (see Materials and Methods). We preselected 865 defined chrY regions (39,126 bp) containing 251 Y-STRs and 772 phylogenetic informative Y-SNPs to cover the entire Minimal reference Y-tree [10]. Primer pairs were designed using DesignStudio by Illumina to create the most optimal panel. They provided us with a list of amplicons and chrY positions that our panel would target in theory. In the results below, this theoretical version of the CSYseq is compared to the output of the CSYseq panel after sequencing: theory versus practice.

In theory, our custom made panel developed by DesignStudio, targets 857 fragments with an average length of 248 bp (range: 225–275 bp). This panel covers 209,248 bp distributed over the euchromatic chrY region and is able to genotype 228 known Y-STRs and 757 phylogenetic informative Y-SNPs. Not all our initially selected Y-STRs and Y-SNPs were included in the amplicon selection made by DesignStudio. This can be due to low primer specificity or undesignable primer sets to avoid Y-SNPs in the primer positions (1000 Genomes as variant source) or a combination of both. The defined amplicon length of 250 bp might be the limiting factor in the selection of the two flanking primers for the assay. In total, 94% of our initial target region is covered by DesignStudio. According to Illumina, a custom design of a TruSeq kit results in at least 70% specificity and 80% coverage of the target regions. Yet, with the CSYseq we reached a coverage of more than 90%.

In practice, after sequencing 130 males, the number of paired-end reads per library was between 346,314 and 2,855,636 (average 818,160 reads). Of the 857 amplicons provided by DesignStudio, 28 amplicons (3.3%) were not sequenced or contained a low depth of coverage, 7 included no known Y-polymorphism, 13 were only partially sequenced (some Y-SNPs were typed, but not the entire Y-STR) and the remaining 809 amplicons provided full sequence reads. The amplicons not containing a known Y-polymorphism is probably a result of low primer design specificity, with the oligos binding on other chrY regions than intended by DesignStudio. Additionally, other genomic regions were targeted due to the sequence homology of several CSYseq primers. Some CSYseq primers aligned on duplicated Y-chromosomal positions or on other chromosomes. The number of aligned reads of all samples and the target chromosome distribution are sorted on alignment percentage with chrY, visualized in a heat map (**S1 Fig**). Obviously, most reads aligned with chrY (67.1%, SD = 9.0%), followed by chrX (4.1%, SD = 0.3%) and chr2 (2.4%, SD = 1.1%). This homology does not affect the results of the CSYseq as our Y-SNP and Y-STR data analysis is sequence-specific which takes flanking and repeat regions into account to filter out homology. The total number of chrY aligned paired-end reads per sample was on average 400,924 reads (range: 67,609–2,573,191 reads). This was nearly twice the depth of coverage (average 250,000 reads) that was necessary to obtain at least 150 single-end reads per amplicon.

### Y-SNPs as evolutionary markers

Y-SNPs are slowly mutating bi-allelic markers used to reconstruct a human phylogenetic tree, to predict ancestral origins and to study evolutionary migration patterns [5,7–9]. Based on the ISOGG YBrowse database (2019–2020), the 841 designed amplicons would target 13,812 known Y-SNPs (http://ybrowse.org/gb2/gbrowse/chrY). As reported by the ISOGG YBrowse database, they can be further divided into 5,927 Y-SNPs with a still unknown phylogenetic position and 7,885 haplogroup-specific Y-SNPs where 30 Y-SNPs have been identified as private SNPs. These haplogroup-specific Y-SNPs define 1,212 unique Y-subhaplogroups (covering 96% of the Minimal Y-tree) [10].

In practice, sequencing 130 males with our CSYseq panel and data analysis with Yleaf [42] successfully enabled the identification of 15,611 Y-SNPs. As reported by the ISOGG YBrowse database (2019–2020), they can be further divided into 6,597 Y-SNPs with a still unknown phylogenetic position and 9,014 evolutionary haplogroup-specific Y-SNPs where 32 Y-SNPs have been identified by ISOGG as private Y-SNPs. The haplogroup-specific Y-SNPs target 1,443 unique Y-subhaplogroups including all main haplogroups (from ‘A’ to ‘T’) divided across the entire phylogenetic tree (**Table 1**). The output of the panel covers 445 haplogroups (97%) of the 458 haplogroups included in the Minimal Y-tree. The 13 Y-subhaplogroups not covered by the panel are B1, C1b1a1a1a1a, I1a2a1a1d1a1a2b1a, K1a, K1b, K2a1a, K2b2, M3, N1a1a1a1a1a6, R1b1a1b1a1a2a1b1a, R1b1a1b1a1a2a2, R1b1a1b1a1a2b3b and S1a2. In total, 129 samples covered the 445 haplogroups and one sample targets only 403 Y-haplogroups. The latter sample was also observed to have the lowest output number of Y-SNPs (7,284) and the lowest chrY alignment percentage (13%). Even though this was a challenging sample, it is still able to target 88% of the haplogroups included in the Minimal Y-tree [10]. In **Table 1**, it can be observed that the CSYseq contains an equal subhaplogroup distribution per main haplogroup compared to the Minimal Y-tree [10]. A complete phylogenetic tree including all CSYseq typed Y-subhaplogroups can be found in **S1 Table** within the Supporting Information file.

**Table 1:** CSYseq Y-SNP and subhaplogroup coverage.

Main Y-SNP haplogroup	Number of subhaplogroups (number of Y-SNPs)		
	Minimal Y-tree [10]	CSYseq in theory	CSYseq in practice
A	23 (45)	40 (274)	41 (370)
B	18 (38)	30 (213)	35 (253)
C	31 (56)	70 (372)	85 (440)
D	8 (22)	37 (109)	43 (135)
E	35 (54)	147 (551)	177 (680)
F	1 (4)	4 (21)	4 (32)
G	24 (45)	76 (282)	102 (347)
H	16 (27)	44 (126)	47 (143)
I	47 (62)	148 (821)	187 (943)
J	29 (72)	103 (820)	122 (983)
K	8 (10)	5 (17)	5 (16)
L	10 (12)	17 (71)	20 (83)
M	12 (29)	16 (48)	14 (51)
N	24 (35)	61 (313)	66 (337)
O	24 (37)	82 (1,424)	92 (1,446)
P	2 (3)	4 (12)	3 (16)
Q	22 (39)	64 (338)	83 (408)
R	106 (143)	233 (1,897)	282 (2,142)
S	12 (18)	17 (51)	17 (56)
T	6 (6)	14 (125)	18 (133)
Total	458 (757)	1,212 (7,885)	1,443 (9,014)

**Fig 1:**
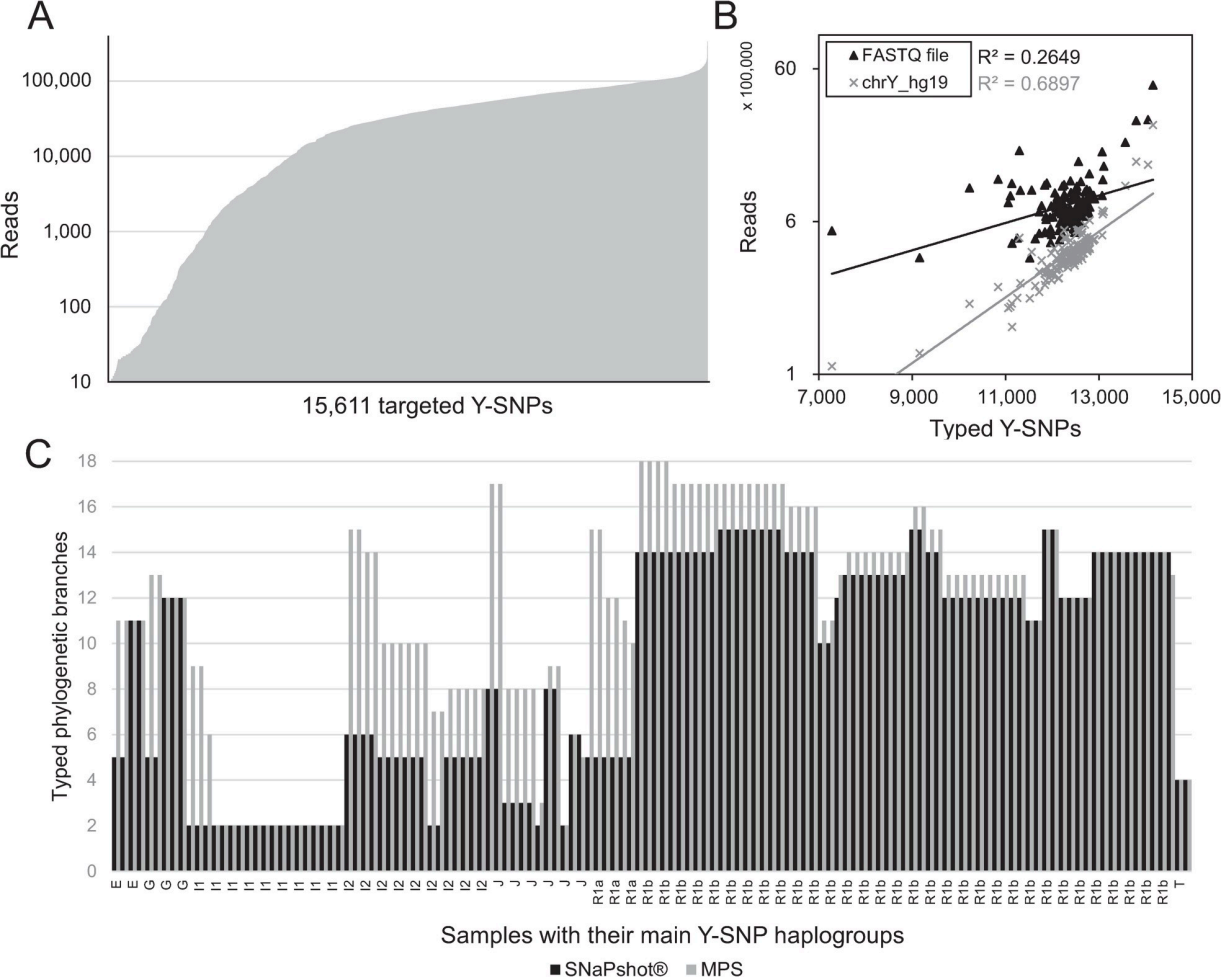
CSYseq targeted Y-SNPs. **A**. The 15,611 genotyped Y-SNPs with number of paired-end reads for all samples (threshold = 10 reads). **B**. Correlation between typed Y-SNPs and the total reads per sample obtained from FASTQ files (▲) and chrY alignment (✖). **C**. Number of typed Y-SNP subhaplogroup branches through SNaPshot-CE (black) compared to MPS (grey) sorted by their main Y-haplogroup.

Targeted Y-SNPs contained between 10 and 5,218 reads per sample with an average of 717 reads. For the 65 non-related samples, the total number of reads per Y-SNP ranged from 10 till 339,189 with 70% between 10,000 and 100,000 reads (**Fig 1A**). On average, there are 12,281 Y-SNPs typed per sample and even the least extensive sample still contained 7,284 well-typed Y-SNPs (**Fig 1B**). The number of typed Y-SNPs per sample was significantly correlated with the total number of reads identified in the FASTQ files (p = 4.33×10^−7^) and the number of reads aligned against chrY (p = 8.85×10^−40^) (**Fig 1B**). This was as expected, since the more reads the sample has in total (FASTQ) or aligned with chrY, the more reads it has per chrY amplicon containing the Y-polymorphisms of interest. Sample quality statistics revealed a slightly significant (at the margin of statistical significance) correlation between typed Y-SNPs with initial chrY concentrations measured before library preparation (p = 1.30×10^−3^) and their degradation index (DI, p = 1.27×10^−2^) (see section ‘CSYseq robustness’, **S2 Fig**). When MPS output is compared to the limited Y-SNP panel typed by the SBE SNaPshot PCR-CE technique used in most laboratories, a successfully deeper Y-SNP subhaplogroup was genotyped for 66% of the samples due to the massive number of typed Y-SNPs (**Fig 1C**). On average, four phylogenetic branches deeper were detracted in which a maximum of ten branches was observed: from ‘R1a1a’ (*R-M198*) with SNaPshot-CE to ‘R1a1a1b1a3a2b2b’ (*R-AM00559)* with MPS. In 32% of the samples, both techniques resulted in the same final derived Y-SNP, but for two samples with subhaplogroups *‘J-M92*’ and ‘*R-L2’*, SNaPshot-CE surpassed MPS in typing one branch deeper. For these latter two cases, the CSYseq panel was able to sequence both final Y-SNPs, but the markers did not pass the selected sequencing criteria of at least 10 reads and the base calling percentage of 90%. This is sample specific and not a limitation of the panel. J-M92 was observed to be typed in 90 samples with an average depth of coverage of 43 reads. And R-L2 was typed in all the other samples with a high average depth of coverage of 1,095 reads.

The female sample revealed output for 399 Y-SNPs, where 205 Y-SNPs (51%) have an unknown phylogenetic position. Detailed Y-SNP analysis revealed no unambiguous haplogroup determination. Although the depth of coverage of these Y-SNPs was above the threshold of 10 single-end reads, they only had a median of 31 reads. Additionally, for all 399 Y-SNPs, the average reads obtained for all male samples were 13 times higher compared to the female sample. This is valuable information for forensic genetics to set a valuable read threshold when DNA mixture analysis needs to be performed.

### Y-STRs as patrilineage markers

Y-STRs provide a high degree of variability due to their fast mutating multi-allelic variations. This makes them highly interesting for population genetics. In theory, the 214 designed amplicons of our CSYseq panel target a total number of 228 Y-STR loci. In practice, after sequencing and Y-STR data analysis with FDSTools, 28 Y-STR loci were excluded from the panel due to not being sequenced (n = 20), low depth of coverage (n = 7) or strong chrX homology which could not be filtered out due to strong sequence similarities (n = 1). This was as expected and can be explained by the 90% primer design success rate of DesignStudio (see before). The excluded Y-STR loci with detailed information are listed in **S2 Table**. Through additional analysis of the high quality sequenced chrY reads with Tandem Repeat Finder (TRF) [43], two novel Y-STR loci were identified which are sequenced by the CSYseq panel. As no information about these specific Y-STRs is yet available in literature or within the ISOGG YBrowse database, they were named CSY1 and CSY2. Further, CSYseq analysis for the double sequenced male sample exposed equal data output and the female sample revealed no output. This indicates that our CSYseq panel is chrY-specific and possible output allele calls as a result of chrX homology were successfully filtered out using our in-house created ‘CSYseq.analYzer’ tool (see Materials and Methods).

In total, the CSYseq panel covers 202 well-targeted Y-STR loci. **Table 2** provides detailed information concerning their repeat motif and discrimination capacity. HGVS nomenclature and Y-chromosome positions of these Y-markers can be found in **S3 Table**. The 202 Y-STRs from the CSYseq panel include 15 Y-STRs from the commercially available CE kits (PowerPlex Y23 and Yfiler Plus): *DYS19*, *DYS389I/II*, *DYS390*, *DYS391*, *DYS392*, *DYS448*, *DYS456*, *DYS635*, *Y-GATA-H4*, *DYS533*, *DYS549*, *DYS570*, *DYS643* and *DYS460*. 17 Y-STRs targeted by the CSYseq are also present in the commercial kits developed for MPS (ForenSeq and PowerSeq): *DYS19*, *DYS389I/II*, *DYS390*, *DYS391*, *DYS392*, *DYS448*, *DYS456*, *DYS460*, *DYS522*, *DYS533*, *DYS549*, *DYS570*, *DYS612*, *DYS635*, *DYS643* and *Y-GATA-H4*. As an internal control, 21 Y-STR loci sequenced by the CSYseq were compared to previously obtained PCR-CE results from our in-house YForGen kit (46 Y-STRs) and commercial Y-kits [44]. We observed that all Y-STR allele calls were in accordance with our previous results, which confirms that the results of MPS are reliable. A total of 188 Y-STRs are simple Y-STRs with one variable repeat motif, while 14 Y-STRs contain a more complex double repeat. For example, ‘*DYS463’* exists of both AAAGG[n] and AAGGG[n] as variable repeat motifs which were easily discriminated using FDSTools. Furthermore, 156 Y-STR loci are single-copy (SC) Y-markers, whereas the other 46 are multi-copy (MC) Y-markers, including three Y-STRs with four loci (*-abcd*). For most MC Y-STRs, it remained difficult to discriminate the different loci due to sequence similarities of the flanking and repeat regions. The results of the MC Y-STR loci with indistinguishable genome alignment were grouped together for further analysis. Equal to CE analysis, if the exact sequence per locus remains unknown, we sort them from short to long Y-STR allele call. This makes it possible to still perform Y-STR mutation analysis using the principle of Parsimony: the least number of changes indicates the most likely event. DNA sequences of all included Y-STRs are publicly available in the ISOGG YBrowse database (http://ybrowse.org/gb2/gbrowse/chrY).

**Table 2:** Detailed information concerning all 202 CSYseq targeted Y-STRs.

Y-STR		Repeat motif			Average repeats	Discrimination capacity
		bp	type	sequence		
*CSY1*		4	S	AGAT[n]	11	0.38
*CSY2*		2	S	TC[n]	25	0.83
*DXYS156*		5	S	TATTT[n]	9	0.04
*DYF371-abcd*		3	S	ACA[n]	12	0.77
*DYF380-ab*		3	S	AAT[n]	10	0.05
*DYF381-ab*		3	S	AAC[8]	8	0.00
*DYF382*		4	S	GGAT[n]	13	0.44
*DYF384-ab*		3	S	CAA[n]	8	0.50
*DYF385-ab*		3	S	TTA[n]	10	0.41
*DYF386-abcd*		3	S	AAT[n]	13	0.74
*DYF389*		4	S	CATC[n]	11	0.40
*DYF391-ab*		4	S	ATAC[n]	9	0.44
*DYF392*		4	S	TTAT[8]	8	0.00
*DYF394*		3	S	AAT[n]	8	0.09
*DYF406*		4	S	TATC[n]	10	0.69
*DYF408-ab*		4	S	ATAG[n]	11	0.82
*DYF409-ab*		4	S	ATAG[n]	12	0.65
*DYF411-ab*		5	S	AAAGG[n]	12	0.69
*DYF412-ab*		5	S	AAATA[n]	13	0.64
*DYS19*	M1—M2	4	X	TATC[n]N[4]TATC[n]	11–3	0.54
*DYS388*		3	S	AAT[n]	13	0.55
*DYS389I*		4	D	TAGA[n]CAGA[3]	10	0.60
*DYS389II*	M1—M2	4	D	TAGA[n]CAGA[n]	11–5	0.52
*DYS390*	M1—M2	4	D	GATA[n]GACA[8]	11–8	0.78
*DYS391*		4	S	TCTA[n]	10	0.54
*DYS392*		3	S	AAT[n]	12	0.58
*DYS413-ab*		2	S	TG[n]	22	0.79
*DYS426*		3	S	GTT[n]	12	0.52
*DYS435*		4	S	TGGA[n]	11	0.09
*DYS436*		3	S	AAC[12]	12	0.00
*DYS442*		4	S	GATA[n]	12	0.55
*DYS445*		4	S	TTTA[n]	12	0.56
*DYS448*	M1—M2	6	X	AGAGAT[n]N[10]AGAGAT[3]N[14]AGAGAT[n]	11–8	0.65
*DYS450*		5	S	TTTTA[n]	9	0.22
*DYS452*	M1—M2	5–10	X	TATAC[n]CATACTATAC[n]	11–2	0.62
*DYS453*		4	S	AAAT[n]	11	0.16
*DYS454*		4	S	AAAT[n]	11	0.13
*DYS455*		4	S	AAAT[n]	11	0.35
*DYS456*		4	S	AGAT[n]	15	0.76
*DYS459-ab*		4	S	AAAT[n]	9	0.64
*DYS460*		4	S	TCTA[n]	11	0.58
*DYS461*		4	S	TCTA[n]	11	0.57
*DYS462*		4	S	ATAC[n]	11	0.50
*DYS463*	M1—M2	5	D	AAAGG[n]AAGGG[n]	6–14	0.74
*DYS467*		4	S	GATA[n]	13	0.60
*DYS470*		3	S	GTT[n]	11	0.07
*DYS474*		3	S	AAC[8]	8	0.00
*DYS475*		3	S	TAA[n]	8	0.06
*DYS476*		3	S	TGA[n]	11	0.14
*DYS477*		3	S	TTG[8]	8	0.00
*DYS478*		3	S	CAC[8]	8	0.00
*DYS480*		3	S	TTA[8]	8	0.00
*DYS484*		3	S	AAT[n]	13	0.32
*DYS490*		3	S	TTA[n]	12	0.20
*DYS492*		3	S	ATT[n]	12	0.39
*DYS497*		3	S	TTA[n]	14	0.45
*DYS507*		4	S	CATA[n]	10	0.18
*DYS510*		4	S	GATA[n]	11	0.52
*DYS511*		4	S	AGAT[n]	10	0.59
*DYS513*		4	S	TCTA[n]	12	0.63
*DYS522*		4	S	ATAG[n]	11	0.63
*DYS523*		4	S	AGAT[n]	13	0.71
*DYS525*		4	S	AGAT[n]	10	0.22
*DYS530*		4	S	AAAC[n]	9	0.29
*DYS531*		4	S	AAAT[n]	11	0.12
*DYS533*		4	S	TATC[n]	12	0.54
*DYS534*		4	S	CTTT[n]	15	0.77
*DYS537*		4	S	TCTA[n]	11	0.61
*DYS538*		4	S	AGAT[n]	11	0.23
*DYS539*		4	S	TAGA[n]	10	0.39
*DYS540*		4	S	TTAT[n]	12	0.38
*DYS541*		4	S	TATC[n]	12	0.63
*DYS542*		4	S	TAGA[n]	12	0.60
*DYS543*		4	S	AGAT[n]	11	0.64
*DYS545*		4	S	TGTT[n]	10	0.52
*DYS549*		4	S	GATA[n]	12	0.60
*DYS552*	M1—M2	4	X	TCTA[n]N[40]TCTA[n]	11–10	0.76
*DYS557*	M1—M2	4	X	TTTC[n]N[3]TTTC[n]	4–16	0.74
*DYS558*		4	S	TTTA[n]	9	0.24
*DYS562*		4	S	ATCT[n]	12	0.73
*DYS565*		4	S	ATAA[n]	12	0.52
*DYS567*		4	S	ATAA[n]	11	0.48
*DYS570*		4	S	CTTT[n]	18	0.73
*DYS573*		4	S	TTTA[n]	10	0.26
*DYS574*		4	S	TTAT[n]	10	0.26
*DYS577*		4	S	ATTC[n]	9	0.03
*DYS581*		4	S	TAGG[n]	8	0.04
*DYS584*		4	S	CAAT[8]	8	0.00
*DYS585*		5	S	TTATG[n]	10	0.50
*DYS587*		5	S	CAATA[n]	11	0.40
*DYS590*		5	S	TTTTG[8]	8	0.00
*DYS593*		5	S	AAAAT[n]	8	0.12
*DYS594*		5	S	AAATA[n]	10	0.30
*DYS595*		5	S	ATTTA[8]	8	0.00
*DYS596*		6	S	GGAGAA[n]	10	0.52
*DYS598*		5	S	TTCTG[n]	8	0.61
*DYS606*		4	S	AAAT[n]	11	0.22
*DYS609*		3	S	TTG[n]	8	0.09
*DYS612*		3	S	TCT[n]	26	0.82
*DYS613*	M1—M2	3	X	ATG[8]N[3]ATG[8]	8–8	0.00
*DYS616*		3	S	TAT[n]	14	0.57
*DYS618*		3	S	TAT[n]	12	0.18
*DYS623*		4	S	GGAT[n]	10	0.12
*DYS624*		4	S	GGAT[n]	9	0.03
*DYS629*		4	S	TATC[n]	9	0.57
*DYS631*		4	S	AATA[n]	10	0.35
*DYS632*		4	S	CATT[n]	9	0.52
*DYS634*		4	S	AAGG[n]	8	0.17
*DYS635*	M1—M2	4	X	TAGA[n]N[8]TAGA[2]N[8]TAGA[n]	10–3	0.72
*DYS637*		4	S	ACAT[n]	11	0.46
*DYS641*		4	S	TAAA[n]	10	0.07
*DYS643*		5	S	CTTTT[n]	11	0.63
*DYS644*		5	S	TTTTA[n]	16	0.75
*DYS645*		5	S	TGTTT[n]	8	0.09
*DYS712*		4	S	AGAT[n]	15	0.85
*DYS715*		4	S	AGAT[n]	13	0.73
*DYS717*		5	S	TGTAT[n]	10	0.31
*DYS723*	M1—M2	4	X	AGAT[n]N[3]AGAT[1]N[3]AGAT[n]	10–6	0.71
*DYS725-abcd*	M1—M2	2–4	X	GT[n]GTCT[n]	20–4	0.83
*TANDEM151*		4	S	TATC[n]	10	0.28
*TANDEM66*		2	S	GT[n]	17	0.28
*TRF10029*		2	S	TG[n]	17	0.53
*TRF10330*		2	S	AC[n]	23	0.77
*TRF10377*		2	S	AC[n]	22	0.74
*TRF10473*		2	S	CA[n]	24	0.61
*TRF10677*		2	S	AC[n]	21	0.42
*TRF10691-ab*	M1—M2	2	X	TG[n]N[10]TG[5]	20–5	0.76–0.00
*TRF10878*		2	S	AC[n]	20	0.66
*TRF11134*		4	S	TATC[n]	7	0.50
*TRF11357*		2	S	TG[n]	17	0.58
*TRF11672*		2	S	AC[n]	12	0.49
*TRF11926*		2	S	GT[n]	19	0.72
*TRF13608*		2	S	TG[n]	22	0.58
*TRF13651*		2	S	AC[n]	22	0.69
*TRF14020*		2	S	TG[n]	15	0.59
*TRF14432*		2	S	AC[n]	20	0.53
*TRF14783*		2	S	GT[n]	20	0.78
*TRF17087*		2	S	TG[n]	17	0.66
*TRF17177*		4	S	ATTT[n]	11	0.26
*TRF17200*		2	S	GT[n]	20	0.68
*TRF3410*		2	S	GA[n]	23	0.76
*TRF4104*		2	S	AC[n]	22	0.68
*TRF4283*		2	S	AG[n]	18	0.59
*TRF4288*		2	S	AC[n]	14	0.12
*TRF4710*		2	S	GT[n]	19	0.52
*TRF4909*		2	S	AC[n]	16	0.44
*TRF5618-a*		2	S	AC[n]	22	0.85
*TRF5618-b*		2	S	TG[n]	17	0.59
*TRF5631*		2	S	TG[n]	18	0.46
*TRF5922*		2	S	AC[n]	18	0.62
*TRF5959*		2	S	TG[n]	20	0.20
*TRF6088*		2	S	GT[n]	23	0.61
*TRF6313*		4	S	AGAT[n]	13	0.70
*TRF6353*		2	S	TG[n]	21	0.78
*TRF6385*		2	S	GT[n]	19	0.18
*TRF6466-ab*		2	S	AC[n]	19	0.76
*TRF6888*		2	S	TG[n]	17	0.63
*TRF7006-ab*		2	S	AC[n]	21	0.69
*TRF7015*		2	S	AT[n]	14	0.68
*TRF7063*		3	S	AAT[n]	13	0.27
*TRF7436*		2	S	TG[n]	18	0.23
*TRF7665*		2	S	GT[n]	21	0.13
*TRF8190*		5	S	TTTTA[n]	12	0.59
*TRF8252*		4	S	AAAT[n]	9	0.50
*TRF8381*		4	S	TAGA[n]	13	0.66
*TRF8424*		5	S	ATATG[n]	9	0.51
*TRF9205-ab*		2	S	AC[n]	20	0.63
*TRF9254*		5	S	AAAAC[n]	11	0.25
*TRF9363*		2	S	TG[n]	18	0.23
*TRF9434*		2	S	AC[n]	23	0.77
*TRF9460*		2	S	GT[n]	19	0.37
*TRF9886*		2	S	AC[n]	19	0.72
*TRF9913*		2	S	TG[n]	18	0.43
*TRF9916*		4	S	TTAT[n]	10	0.14
*YCAII-ab*		2	S	CA[n]	21	0.70
*Y-GATA-A10*		4	S	ATCT[n]	13	0.57
*Y-GATA-H4*		4	S	CTAT[n]	11	0.58

*Note*: -a, -b: multi-copy Y-STRs; -M1, -M2: Separate variable motifs within complex Y-STRs; S: simple, X: complex, D: compound; Grey within repeat sequence: interruptions (N) and non-variable motifs.

The CSYseq targets 57 di-, 38 tri-, 83 tetra-, 22 penta- and 2 hexanucleotide repeats. For autosomal DNA analysis, the two genuine allele calls of dinucleotide STRs can be difficult to interpret due to their stutter fragments. For Y-chromosomal STR analysis, this is different as it mostly results into one allele call due to its haploid nature. For the single-copy dinucleotide Y-STRs, stutter fragments and the true allele call can easily be identified using FDSTools. But the panel does include six multi-copy dinucleotide Y-STRs that cannot be distinguished by sequence variance in the flanking regions. These Y-STRs resulted in multiple stutter peaks. Therefore, a more complex separation of stutter alleles and genuine heterozygous alleles was necessary. The reported difficulties with these Y-STR stutters were taken into account within the CSYseq.analYser file. This file sorts the sequences according to their number of reads to additionally filter out all stutters. An example of the different allele and stutter output combinations to interpret multi-copy dinucleotide YCAII-ab within our sample is provided in **S4 Table**.

**Fig 2:**
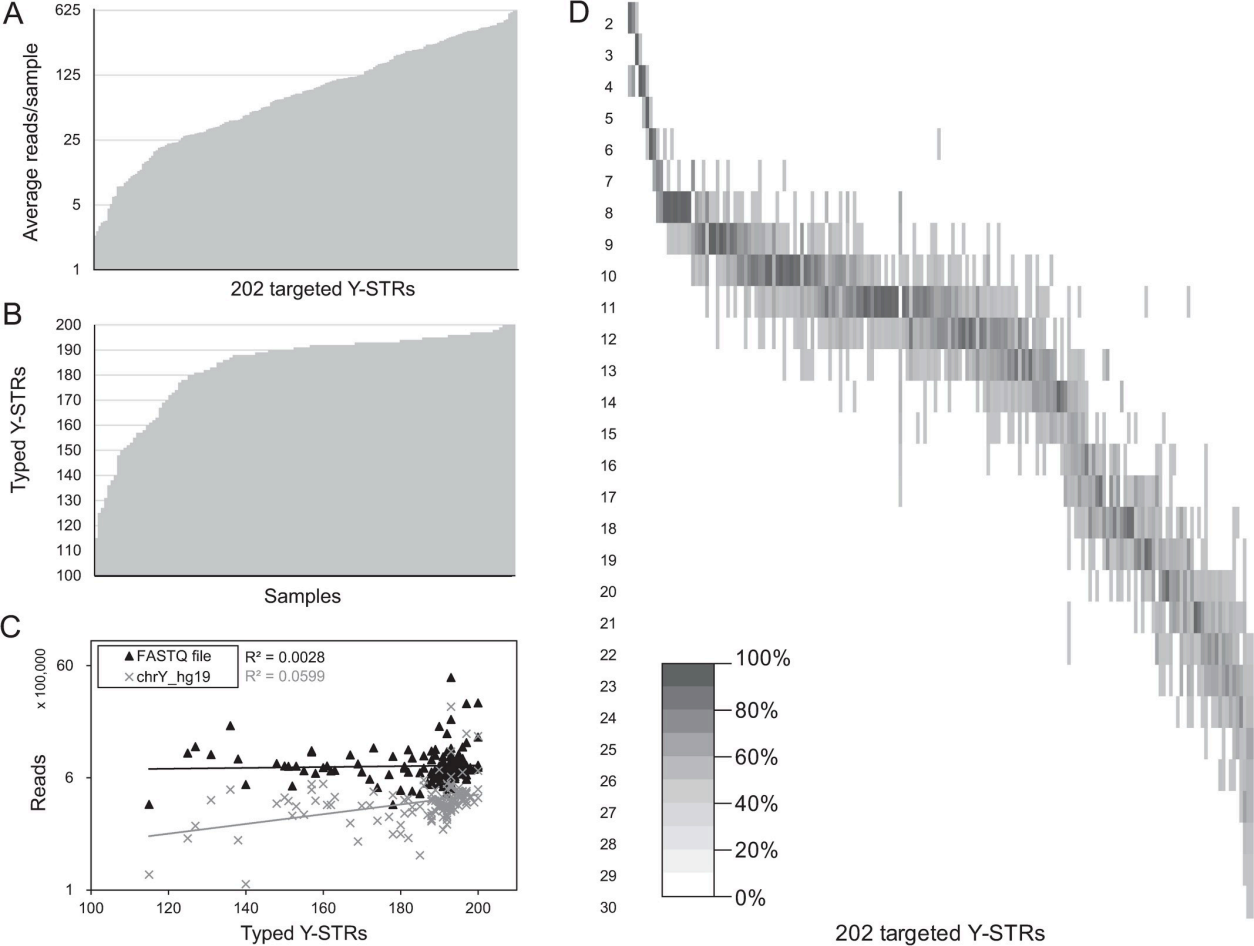
CSYseq targeted Y-STRs. **A**. The average number of reads of the 202 targeted Y-STR loci of the CSYseq panel. **B**. The number of typed Y-STRs per sample. **C**. Correlation between the typed Y-STRs per sample and the total number of reads per sample obtained from FASTQ files (▲) and chrY alignment (**×**). **D**. A heatmap visualizing the allele ranges and frequencies per Y-STR.

The average number of reads per Y-STR locus was 150 reads, ranging from 5 (*DYS448*) to 619 reads (*TRF17200*) (**Fig 2A**). Only 11 Y-STR loci had an average number of reads below 10, though eight of them were genotyped for the majority of the samples. These markers may have insufficient coverage for challenging forensic samples, but this needs to be confirmed by future research. However, they can still be interesting to include into the panel for genetic-genealogy purposes and mass-screening for forensic familial searching. 137 Y-STRs are well-typed in more than 90% of the samples, 54 Y-STRs in 60 to 90% and 11 Y-STRs in 30–60% of the samples. The number of Y-STRs typed per sample is visualized in **Fig 2B**. On average, there are 184 Y-STRs typed per sample and even the least extensive Y-haplotype still contained 115 well-typed Y-STRs. There was no significant correlation between the number of typed Y-STRs and the number of reads within their FASTQ file (**Fig 2C**). However, the number of typed Y-STRs was observed to correlate significantly with the number of reads aligned against chrY (p = 6.90×10^−3^). Sample quality statistics revealed a slightly significant (at the margin of statistical significance) correlation between typed Y-STRs and the initial chrY concentrations measured before library preparation (p = 2.80×10^−2^), but not with the DI (see section ‘CSYseq robustness’, **S2 Fig**).

All 202 Y-STR loci were investigated in detail using GenAlEx to determine the allele call frequencies with allele ranges (**Fig 2D**), discrimination capacity, and average repeat sizes (**Table 2**). The 14 Y-STR loci having multiple variable repeat units were divided into -M1 and -M2. The smallest variable repeat size contained only two repeats (*DYS452-M2*, *DYS635-M2* and *DYS19-M2*), while the largest repeat number observed contained 30 repeats (*DYS612*). Detailed double repeat sequence variability with their allele call frequencies for the 14 variable complex and compound Y-STRs can be found in **S5 Table** within the Supporting Information file. Average discrimination capacity was 0.69 for complex and compound Y-STRs and 0.44 for simple Y-STRs. For 11 Y-STRs, no allele diversity was observed between the samples included in this study, wherefore consequently a discrimination capacity of zero was calculated.

**Fig 3:**
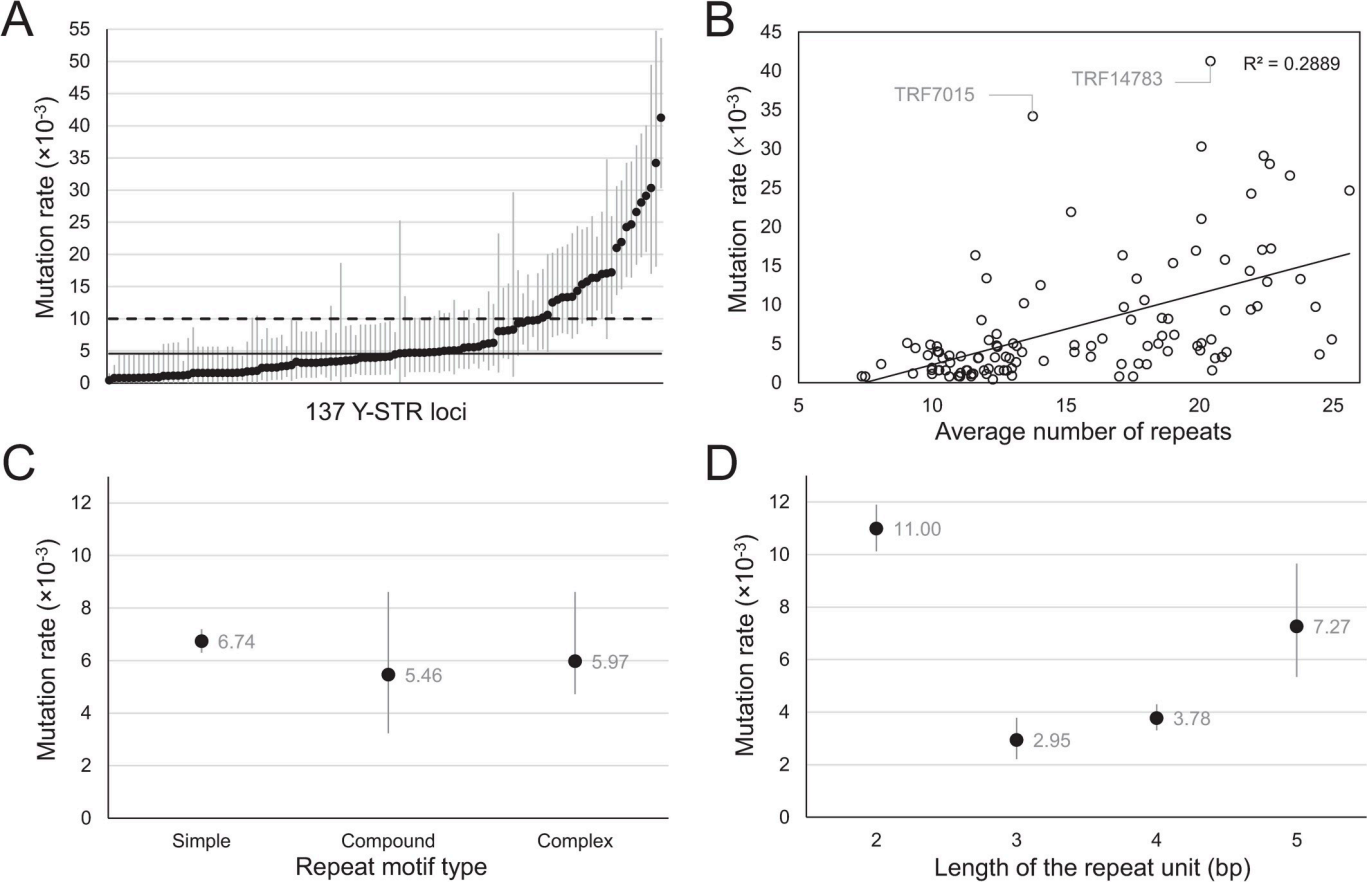
CSYseq Y-STR mutation analysis. **A**. Individual Y-STR mutation rates with their 95% CIs. The RM Y-STR treshold (10^−2^ mpg, dashed line) and average mutation rate (4.57×10^−3^ mpg, black line) are indicated. **B**. Positive significant correlation between the Y-STR mutation rate and the average number of repeats. **C**. The average Y-STR mutation rates of the different repeat motif types (simple, compound and complex). **D**. Average mutation rates per length of the repeat unit (bp).

Y-STR mutation analysis was conducted through Y-haplotype comparison between male relatives within the genealogical pairs and deep-rooting pedigrees. A detailed overview of the mutation statistics per Y-STR loci are listed in **Table 3**. The number of generations covered per Y-STR loci are on average 1,083 meioses and fluctuated between 218 and 1,279 meioses. This fluctuation can be explained by the fact that some Y-STR markers were not successfully typed in all samples. A total number of 910 Y-STR differences was observed over 214,859 allele transfers (**Table 3**). In total, 759 one-step, 98 two-step and 53 multi-step differences were observed. For 66 Y-STRs, no allele call differences within the sequenced genealogical pairs were observed. The mutation rates of the other 136 Y-STRs are listed with their 95% confidence interval (CI) in **Table 3** and visualized in **Fig 3A**. An overall average mutation rate of 4.57×10^−3^ mpg (95% CI: 4.29×10^−3^–4.86×10^−3^) was observed for the CSYseq panel. When we exclude the Y-STRs without an observed mutation in our study, an average mutation rate of 6.64×10^−3^ mpg was obtained with a minimum of 4.15×10^−4^ mpg (*DYS371-abcd*) and a maximum of 4.13×10^−2^ mpg (*TRF14783*). The mutating Y-STRs can be subdivided into 15 slow mutating Y-STRs (<10^−3^ mpg), 68 moderate mutating Y-STRs (≥10^−3^ to <5×10^−3^ mpg), 27 fast mutating Y-STRs (≥5×10^−3^ to <10^−2^ mpg) and 26 rapid mutating Y-STRs (≥10^−2^ mpg, **Fig 3A**, red line) [45]. The individual mutation rates of 101 Y-STRs were compared to literature [14,17,46–48]. In total, 95% of these Y-STRs were in accordance with literature, which means that a significantly different mutation rate was observed for only five Y-STRs (*DYS390*, *DYS490*, *DYS525*, *DYS606* and *DYS612*). For the influencing molecular factors, a significant positive correlation between the individual Y-STR mutation rates with the average allele size (number of repeats) (p = 8.34×10^−10^) was observed (**Fig 3B**). Mutability rates had no significant difference between simple, compound or complex repeat Y-markers (**Fig 3C**). Further, significant differences were identified in the mutation rates between di-, tri-, tetra- and pentanucleotide Y-STRs, but no significant difference between tri- and tetranucleotide Y-STRs nor a linear correlation was observed (**Fig 3D**).

**Table 3:** CSYseq Y-STR mutation analysis.

	Y-STR differences										Y-STR mutation rate (×10^−3^)					
Y-STR	1	2	3	4	5	6	7	total		m	mpg	95% CI	mpg ref.			
*DYF371-abcd*	2							2		4,822	0.41	0.05–1.50	1.51	1		
*DYS435*	1							1		1,279	0.78	0.02–4.35	1.00	1		
*DYS643*		1						1		1,279	0.78	0.02–4.35	1.21	4		
*TRF9913*	1							1		1,279	0.78	0.02–4.35	0.42	3		
*TRF5618-b*		1						1		1,279	0.78	0.02–4.35				
*DYF384-ab*	2							2		2,558	0.78	0.09–2.82				
*TRF11134*	1							1		1,237	0.81	0.02–4.50	0.42	3		
*DYS562*	1							1		1,218	0.82	0.02–4.57	0.51	3		
*DYS538*	1							1		1,202	0.83	0.02–4.63	0.39	1		
*DYS461*	1							1		1,155	0.87	0.02–4.81	0.99	1		
*DYS452*	1							1		1,129	0.89	0.02–4.93	4.02	1		
*DYS445*	1							1		914	1.09	0.03–6.08	2.16	1		
*DYS618*	1							1		906	1.10	0.03–6.13	0.40	1		
*DYS641*	1							1		887	1.13	0.03–6.27	0.39	1		
*DYS565*	1							1		881	1.14	0.03–6.31	2.09	1		
*DYF391-ab*	3							3		2,558	1.17	0.24–3.42	0.35	3		
*DYS454*	1							1		795	1.26	0.03–6.99	0.48	1		
*DYS388*	1							1		642	1.56	0.04–8.65	0.43	1		
*DYS510*	1	1						2		1,279	1.56	0.19–5.64	5.99	1		
*DYS539*	2							2		1,279	1.56	0.19–5.64	1.00	1		
*DYS541*	2							2		1,279	1.56	0.19–5.64	3.92	1		
*Y-GATA-A10*	2							2		1,279	1.56	0.19–5.64	3.32	1		
*Y-GATA-H4*	2							2		1,279	1.56	0.19–5.64	3.01	4		
*DYS462*	2							2		1,279	1.56	0.19–5.64	2.65	1		
*DYS467*	2							2		1,279	1.56	0.19–5.64	5.21	3		
*YCAII-ab*	1				1			4	(2–6)	2,558	1.56	0.43–4.00	0.57	2		
*DYF385-ab*	3							3		1,876	1.60	0.33–4.67	2.51	4		
*DYF380-ab*	4							4		2,436	1.64	0.45–4.20	0.38	1		
*DYS492*	2							2		1,120	1.79	0.22–6.44	0.39	1		
*TRF7063*	1							1		545	1.83	0.05–10.18	0.90	3		
*DYS573*		1						1		529	1.89	0.05–10.49	0.41	1		
*TRF7436*	2	1						3		1,279	2.35	0.48–6.84	0.29	3		
*TANDEM66*	2							2		843	2.37	0.29–8.54	0.57	3		
*DYS634*	3							3		1,258	2.38	0.49–6.95	0.42	1		
*TRF5922*	3							3		1,237	2.43	0.50–7.07	0.33	3		
*DYS543*	3							3		1,170	2.56	0.53–7.47	7.10	1		
*DYF386-abcd*	4		1					6	(5–7)	2,283	2.63	0.97–5.71	6.02	1		
*DYS497*	2							2		719	2.78	0.34–10.01				
*DYS552*	4							4		1,279	3.13	0.85–7.99	2.69	1		
*DYS391*	4							4		1,279	3.13	0.85–7.99	2.53	4		
*DYS533*	4							4		1,279	3.13	0.85–7.99	3.68	4		
*TRF6313*	4							4		1,279	3.13	0.85–7.99	1.63	3		
*DYF409-ab*	7	1						8		2,504	3.19	1.38–6.29	2.31	3		
*DYS513*	4							4		1,237	3.23	0.88–8.26	6.09	1		
*TRF7006-ab*	2	1						3		908	3.30	0.68–9.62	1.07	3		
*DYS644*	2							2		600	3.33	0.40–11.99	3.22	1		
*DYF412-ab*	3		1					5	(4–6)	1,498	3.34	1.08–7.77				
*DYS606*	1							1		296	3.38	0.09–18.68	0.40	1	★	
*DYS460*	4							4		1,151	3.48	0.95–8.87	5.82	4		
*DYS511*	4							4		1,137	3.52	0.96–8.98	1.52	1		
*TRF10691-ab*	2	3	1					7	(6–8)	1,934	3.62	1.46–7.44	0.62	3		
*DYF406*	5							5		1,279	3.91	1.27–9.10	3.82	1		
*DYS715*	5							5		1,279	3.91	1.27–9.10	3.35	3		
*TRF10677*	5							5		1,279	3.91	1.27–9.10	1.08	3		
*DYS456*	5							5		1,279	3.91	1.27–9.10	4.41	4		
*TANDEM151*	5							5		1,248	4.01	1.30–9.32	1.37	3		
*TRF6385*	4	1						5		1,237	4.04	1.31–9.41	0.33	3		
*DYS557*	5							5		1,211	4.13	1.34–9.61	3.8	1		
*DYF408-ab*	7						1	11	(8–14)	2,485	4.43	2.21–7.91	1.57	3		
*TRF8190*	1							1		218	4.59	0.12–25.29	1.05	3		
*DYS525*	3							3		646	4.64	0.96–13.51	0.98	1	★	
*DYS723*	6							6		1,279	4.69	1.72–10.18	3.03	5		
*TRF8381*	5	1						6		1,279	4.69	1.72–10.18	4.54	3		
*TRF5959*	6							6		1,279	4.69	1.72–10.18	0.36	3		
*TRF9363*	6							6		1,279	4.69	1.72–10.18	0.45	3		
*DYS549*	6							6		1,261	4.76	1.75–10.33	3.33	4		
*DYS534*	6							6		1,250	4.80	1.76–10.42	6.51	1		
*DYS389I*	5							5		1,030	4.85	1.58–11.29	2.72	4		
*TRF4710*	6							6		1,200	5.00	1.84–10.85	0.53	3		
*DYS635*	4		1					6	(5–7)	1,191	5.04	1.85–10.93	4.21	4		
*DYS463*	4							4		790	5.06	1.38–12.91	1.51	1		
*DYS459-ab*	4	5						9		1,774	5.07	2.32–9.61	2.67	1		
*DYS442*	7							7		1,279	5.47	2.20–11.24	9.78	1		
*CSY2*	7							7		1,261	5.55	2.23–11.40				
*TRF9205-ab*	6	8						14		2,522	5.55	3.04–9.30	1.09	3		
*DYS389II*	7							7		1,237	5.66	2.28–11.62	4.33	4		
*TRF6466-ab*	4	9	1					15	(14–16)	2,496	6.01	3.37–9.89	0.34	3		
*TRF9886*	5							5		816	6.13	1.99–14.24	0.36	3		
*DYS542*	8							8		1,279	6.25	2.70–12.29	5.45	1		
*DYS490*	3							3		374	8.02	1.66–23.26	0.40	1	★	
*TRF11357*	8	2						10		1,237	8.08	3.88–14.82	0.41	3		
*TRF9460*	9							9		1,101	8.17	3.74–15.46	0.78	3		
*DYS390*	2							2		241	8.30	1.01–29.65	2.08	4	★	
*TRF7665*	8	1						9		971	9.27	4.25–17.52	0.53	3		
*DYS413-ab*		7	1	2	1	2		24	(13–35)	2,558	9.38	6.02–13.93	1.22	3		
*TRF6888*	12							12		1,237	9.70	5.02–16.88	0.36	3		
*DYS725-abcd*	2	6	7	1	1	1		49	(36–62)	5,041	9.72	7.20–12.83	0.99	3		
*TRF13608*	11	1						12		1,224	9.80	5.08–17.06	0.77	3		
*DYS523*	11	1						12		1,179	10.18	5.27–17.71	2.53	3		
*TRF5631*	9							9		849	10.60	4.86–20.03	0.33	3		
*TRF4288*	13				1			16	(14–18)	1,279	12.51	7.17–20.24	0.50	3		
*TRF6088*	16							16		1,237	12.93	7.41–20.92	1.46	3		
*TRF10473*	17							17		1,279	13.29	7.76–21.20	0.85	3		
*DYS570*	14	2						16		1,200	13.33	7.64–21.56	11.35	4		
*TRF11672*	11	1						12		896	13.39	6.94–23.28	0.26	3		
*TRF4104*	9		2					13	(11–15)	907	14.33	7.65–24.39	1.67	3		
*TRF11926*	18	1						19		1,238	15.35	9.26–23.86	0.65	3		
*TRF6353*	18	2						20		1,269	15.76	9.65–24.24	1.24	3		
*DYF411-ab*	16	5		4	1			34	(26–42)	2,082	16.33	11.34–22.75				
*TRF17087*	11		1	1				15.5	(13–18)	949	16.33	8.87–25.94	0.38	3		
*TRF14432*	18							18		1,063	16.93	10.07–26.63	0.98	3		
*TRF5618-a*	7							7		411	17.03	6.87–34.78	1.62	3		
*TRF3410*	19	3						22		1,279	17.20	10.81–25.93	1.76	3		
*TRF10878*	24	2						26		1,238	21.00	13.76–30.62	0.63	3		
*DYS712*	21	3	2					28	(26–30)	1,279	21.89	14.60–31.49	30.30	5		
*TRF10377*	29	2						31		1,279	24.24	16.53–34.23	0.74	3		
*DYS612*	2	6	1	1				30.5	(28–33)	1,237	24.66	16.42–34.44	14.50	1	★	
*TRF10330*	27	7						34		1,279	26.58	18.48–36.95	1.58	3		
*TRF9434*	34	1						35		1,248	28.04	19.61–38.79	0.93	3		
*TRF13651*	29	7						36		1,237	29.10	20.46–40.06	1.13	3		
*TRF17200*	14	1						15		495	30.30	17.06–49.49	0.59	3		
*TRF7015*	12	1	1					14.5	(14–15)	424	34.20	18.17–54.78	0.44	3		
*TRF14783*	14	2	9	5	1			49.5	(31–68)	1,200	41.25	30.36–53.63	0.79	3		
66 Y-STR loci without allele differences								0		66,972						
Total	759	98	29	14	6	3	1	982	910–1,055	214,859	4.57	4.29–4.86				

*Note*: *m*: *number of meioses; CI*: *confidence interval; -a*, *-b*: *multi-copy Y-STRs; 1–7*: *one- to 7-step Y-STR differences; within brackets*: *under- and overestimation when larger multistep mutations are present;*★: *significant difference with p<0*.*05; mpg references*: *1 =* [14]*; 2 =* [17]*; 3 =* [47]*; 4 =* [48]*; 5 =* [46]

Through detailed mutation analysis of complex and compound Y-STRs, it was observed that Y-STR differences occurred more frequently within the longest repeat sequence. For example, in *DYS725-abcd*, which has a compound repeat structure being GT[n]GTCT[n], the average number of repeats is respectively 20 and 4, and the observed number of mutations per motif is 32 and 17. Furthermore, we observed that three markers (*TRF10691*, *DYS463* and *DYS725*) showed allele call differences in five genealogical pairs for both variable motifs at the same time. For three couples, these differences were found on *DYS725* (NC_000024.10:g.24738202) e.g. one relative had GT[19]GTCT[4], while the other contained GT[20]GTCT[5] which reveals two independent one-step mutations in parallel. The other two genealogical pairs contained both a parallel mutation which would have remained hidden through CE as the two mutations resulted in the same allele call: 22 repeats for *DYS463* (NC_000024.10:g.7775468) with AAAGG[7]AAGGG[15] ↔AAAGG[8]AAGGG[14] and 25 repeats for *TRF10691* (NC_000024.10:g.15550131) with TG[21]N[10]TG[4] ↔TG[20]N[10]TG[5].

Through comparison analysis of the 202 Y-STR loci, it was possible to distinguish all non-related and related males, providing 130 unique Y-haplotypes. Using the Y-STR differences observed over the 136 mutating loci, the CSYseq succeeded in making a distinction between all paternally related males. On average, they were separated by 18 generations and discriminated by 13 Y-STR changes. A minimum number of four Y-STR differences was observed for a couple separated by 18 meioses, whereas a maximum of 22 Y-STR changes for two couples could be observed separated by 21 and 29 meioses. No significant correlation was observed between the number of generations and the number of mutations. This can be explained by the inclusion of fast and rapid mutating Y-STRs in the CSYseq panel and by the occurrence of back and parallel mutations which increases with the generational distance within genealogical pairs [23].

### CSYseq robustness

A schematic overview of the MPS library quality and chrY data analysis steps is provided in **S2A Fig**. The TruSeq Custom Amplicon Low Input kit (Illumina, San Diego, CA, USA) recommends a DNA input of 10 ng and DNA concentration of 2.5 ng/μl [49]. The chrY DNA input concentration of all sequenced samples measured using PowerQuant qPCR was between 1.75 and 17.58 ng/μl (average 5.69 ng/μl) with a degradation index (DI) from 0.90 to 4.79 (average 1.92; **S2B Fig**). No significant correlation between chrY concentration with DI could be observed. Five samples did not fulfil the recommended input concentration from which two samples had a DI exceeding the manufacturer’s threshold of 2 [50]. The samples encountering the highest DI (4.79) and the lowest chrY concentration (1.75 ng/μl) are respectively indicated by the labels ‘d1’ and ‘c1’ throughout **S2 Fig**.

Library preparation quality control measured by the 2100 BioAnalyzer indicated a library peak size for all samples between 357 and 397 bp (average 380 bp) and a library concentration between 0.01 and 8.82 ng/μl (average 1.6 ng/μl; **S2C Fig**). BioAnalyzer library concentrations showed a significant correlation with the initial identified chrY concentrations (p = 4.09×10^−3^), but not with the DI. Additionally, normalized KAPA SYBR qPCR Ct values (**S2D Fig**) also revealed a significant correlation with the chrY concentrations (p = 1.27×10^−6^), but are only slightly significant with the DI (p = 0.006, R^2^ = 0.062). As expected, normalized KAPA qPCR Ct values correlated significantly with the BioAnalyzer library concentrations (p = 1.24×10^−10^, R^2^ = 0.293). The number of FASTQ file reads per library output after sequencing (**S2E Fig**) did not significantly correlate with both the initial chrY concentrations and the DI due to library normalization.

FASTQC software [51] flagged 63 samples with high per sequence base quality, meaning that, for both paired-end reads, the lower quartile of the first 150 bp did not have a FASTQC quality Phred score below 20. For all samples, the read position where FASTQC Phred scores went below 20 ranged from 85 to 278 bp (average 172 bp; **S2F Fig**). Again, only a slightly significant correlation could be observed with the initial chrY concentrations (p = 2.54×10^−3^), but not with the DI. Besides, the percentage of read alignment against GRCh37/hg19 reference genome and chrY also turned out to be only significant with the input chrY concentrations (p = 4.33×10^−3^ and 6.16×10^−4^) and not the DI. Remarkable was that both samples with the highest DI (4.79 and 4.69) showed a high and low quality. The FASTQC Phred score below 20, defining low per sequence base quality, are respectively at 258 bp and 104 bp and they have a chrY alignment of 61% and 36%. However, a high number of Y-markers (12,709 and 12,565 Y-SNPs; 186 and 192 Y-STRs) could still be sequenced (**S2F and S2G Fig**). In general, the number of typed Y-SNPs using Yleaf (**S2G Fig**) showed a slightly significant correlation with the input chrY concentrations (p = 1.30×10^−3^) and DI (p = 1.27×10^−2^). Yet the number of typed Y-STRs using FDSTools (**S2H Fig**) only turned out to be slightly significant with the input chrY concentrations (p = 2.80×10^−2^), but not the DI. Therefore, the success rate of the CSYseq panel was not clearly observed to be influenced by the initial chrY concentration or degradation index of a sample.

Further, we focus on sample d1 with the highest degradation index (4.79) and c1 with the lowest initial chrY concentration (1.75 ng/μl), both indicated throughout **S2 Fig**. We observed that they both encountered low concentrations after library preparation measured by the BioAnalyzer and KAPA qPCR (**S2C and S2D Fig**). Surprisingly, they both contained relatively high paired-end read outputs of respectively 1,065,926 and 939,462 reads. The paired-end read alignment against the GRCh37/hg19 reference genome differed strongly with respectively 85% and 25% and for chrY alignment this was respectively 61% and 16%. Consequently, d1 contained an overall higher FASTQC quality Phred score (from 258 bp below 20) compared to c1 (from 85 bp below 20; **S2E and S2F Fig**). As a result, the number of typed Y-SNPs was slightly higher for d1 (12,706 Y-SNPs; 81%) compared to c1 (11,139 Y-SNPs; 71%; **S2G Fig**). But, remarkably, with the high number of typed Y-SNPs, they both still resulted in well-typed deep Y-subhaplogrouping. The CSYseq kit even added eight branches in c1 from ‘I2a1b1’ with CE to ‘I2a1b1a2b1a1a1’. Additionally, a high number of typed Y-STRs for both d1 and c1 was still possible, respectively being 186 Y-STRs (92%, average = 245 reads per Y-STR) and 182 Y-STRs (90%, average = 51 reads per Y-STR; **S2H Fig**).

## Discussion

In this study, we present the ‘CSYseq’ panel which allows the identification of 9,014 phylogenetic Y-SNPs and 202 interesting Y-STRs through massive parallel sequencing (MPS). We sequenced one female sample and 130 males from the Low Countries (Belgium or the Netherlands) distributed over 65 different paternal pedigrees. This enabled us to analyze and investigate all Y-polymorphisms included in the CSYseq panel on their ease of interpretation, depth of coverage, discrimination power, mutability and chrY specificity.

### Y-SNPs as evolutionary markers

Y-SNPs enable the reconstruction of a well-preserved male phylogenetic tree. Neighboring populations represent a comparable evolutionary haplogroup distribution, while different continents can exhibit large differences. Y-SNPs are evolution markers which are more frequently present within specific geographical regions, for example ‘R-M269’ to West-Europe, ‘E1b1b’ to North-Africa and ‘Q’ to America [6,52–54]. This is of course without taking recent migration into account. The CSYseq panel successfully enabled the identification of 15,611 Y-SNPs, which is even more than the panel was estimated to target in theory due to primer homology. In total, 9,014 Y-SNPs are defined by the ISOGG YBrowse Database (2019–2020) as haplogroup-specific Y-SNPs targeting 1,443 evolutionary Y-subhaplogroups (**Table 1**). Since the Minimal Y-tree by Van Oven *et al*. is commonly used in Y-SNP genotyping, a large coverage is desired which facilitates Y-SNP genotyping [10]. With our CSYseq panel, 445 out of 458 subhaplogroups present in the Minimal Y-tree were genotyped, resulting in a successful coverage of 97%. In addition, 998 additional subhaplogroups were covered using the CSYseq panel. The number of phylogenetic Y-SNPs typed with the CSYseq is more than ten times higher than the current most extensive Y-SNP MPS kit on the market, namely the Ion AmpliSeq HID Y-SNP Research Panel v1 (Thermo Fisher Scientific) with 859 phylogenetic Y-SNPs [41]. Consequently, it analyzes 56% less Y-haplogroups (n = 640) than the CSYseq panel (n = 1,443). This large number of Y-SNPs spread over the euchromatic region of the Y-chromosome is interesting when we have to analyze challenging or degraded samples. Additionally, the CSYseq targets all main haplogroups (‘A’ to ‘T’) divided across the entire human Y-chromosome phylogenetic tree. This custom-made panel can therefore serve as a powerful tool to identify paternal evolutionary lineages and provide more information about males with any biogeographical background around the world. It is interesting to note that every main subhaplogroup present in our population (Belgium and the Netherlands) contained a high number of typed Y-SNPs in our sample (**S3 Fig**). Although sampling males with other biogeographical backgrounds still remains necessary, we are confident by the discrimination power of the CSYseq due to its high Y-SNP coverage across all main haplogroups of the human Y-chromosome phylogeny. In addition, about 6,597 Y-SNPs targeted by the CSYseq currently have no available information on their biogeographic paternal ancestry. As the list of Y-SNPs in the ISOGG index is constantly being updated with newly identified Y-SNPs, even more haplogroup-specific Y-SNPs will be identified using our CSYseq when more extensive geographic sampling will be performed in the future.

As a result of MPS, identification and mapping of Y-SNPs on the phylogenetic tree in population studies is facilitated as more Y-SNPs can be targeted and a large sample input (from a specific population) can be sequenced. Additional population data of newly discovered Y-SNPs still remains necessary to obtain as Y-SNP expansion gives rise to some complications. First, universal names for Y-SNPs are non-existing, meaning that equality and comparisons between studies and different phylogenetic trees remain extremely difficult to achieve [55]. For example, the Y-SNP *R-L11* has also been described as *R-S127* and *R-PF6539*. With the CSYseq, we present a universally exchangeable set of interesting Y-markers which can provide a basis for a uniform nomenclature between Y-SNP population studies worldwide. Second, many Y-SNPs are already mapped in a phylogenetic tree despite the lack of large-scale population data. Consequently, their position on the phylogenetic tree is deceitful and cannot be used as additional biogeographical background information [2,55,56]. For 183 Y-SNPs, a combination between ancestral and derivative alleles was observed within our samples indicating that these markers exhibit larger diversity within the population of the Low Countries (Belgium and the Netherlands). Herein, many unlisted, unknown Y-SNPs and Y-SNPs associated with multiple haplogroups are incorporated due to which they may not be allocated to a specific haplogroup yet be private to some families. The distribution of ancestral and derivative calls within a specific Y-SNP can serve as valuable information for population genetics since Y-SNPs with larger diversity have a higher discrimination power between individuals.

### Y-STRs as patrilineage markers

Y-STRs are commonly used DNA polymorphisms to find distant or close relatives through patrilineage identification in interdisciplinary research fields. The current most extensive MPS kit for Y-STR haplotyping is the ForenSeq DNA Signature Prep kit (Illumina, 2015) targeting 24 Y-STRs [37]. As 24 Y-STRs is even smaller than the commercially available PCR amplification kit Yfiler Plus, including 27 Y-STR loci for fragment analysis by CE, the development of a more extensive MPS kit, sequencing more Y-STR markers, was definitely required. Our custom-made CSYseq panel successfully targets 202 Y-STR loci, where 15 Y-STR loci are present in today’s commercial PCR amplification Y-kits (PowerPlex Y23 and Yfiler Plus) and 17 Y-STR loci in commercial MPS kits (ForenSeq and PowerSeq). This is useful for chrY comparison with, for instance, the YHRD reference database where haplotypes from all over the world are gathered [57]. However, further inclusion of the other forensic commercially available Y-STR loci in the CSYseq will increase compatibility with the existing Y-haplotype kits. Next, the CSYseq output is highly chrY-specific as Y-STRs with primer homology on other chromosomes were successfully excluded by our in-house ‘CSYseq_analYser’ due to sequence or allele call differences. Only one Y-STR was excluded from the panel as it exhibited homology with chrX to such an extent that an extreme high number of reads was obtained for the homology allele calls. On average, the 202 Y-STRs within the CSYseq panel were well-typed in 90% of the samples, containing a high average depth of coverage from 150 reads. Unfortunately, for the majority of MC Y-STRs, FDSTools was still unable to distinguish between the different loci due to the extreme sequence similarity of the flanking regions for both loci. This causes non-distinguishable MC Y-STR loci to have a more complex separation of stutter alleles and genuine heterozygous alleles. However, the FDSTools software did succeed in genotyping more complex repeat structures. Moreover, the reported difficulties with these Y-STR stutters were taken into account within the CSYseq.analYser file. This file sorts the sequences according to their number of reads to additionally filter out all stutters. This is especially important for the dinucleotide Y-STRs, which result in multiple stutter peaks. Fortunately, no Y-STR loci had to be excluded due to unsuccessful genotyping caused by the complexity of the repeat structure, as was the case with previous STRaitRazor genotyping [58].

### Y-STR discrimination power

The CSYseq includes 46 multi-copy Y-STRs and 26 RM Y-STR loci, which strongly increases the discrimination power of the panel. Our detailed population study revealed no variation for 11 Y-STRs, which was also observed before [59]. Their rather small allele size could explain the low variability. Moreover, our sample consisted exclusively of men from the Low Countries, which means that Y-marker variability in different populations cannot be excluded. Next, 25 Y-STRs from the CSYseq panel exhibited a high degree of variation (discrimination capacity >0.75) in our population. The most discriminating Y-STR in our panel is *DYS712*, which ranges from 11 to 22 repeats with a discrimination capacity of 0.85. This is presumably a result of the rapidly mutating nature of this locus (2.19×10^−2^ mpg). Additionally, the compound and complex Y-STRs contain a higher discrimination capacity of 0.7 compared to 0.4 for the simple Y-STRs. Further, these markers provide the advantage to discriminate males containing equal total allele sizes through detailed repeat motif analysis. For instance, within the complex Y-STR *DYS552*, this resulted in twice the number of different allele calls typed with MPS (n = 12) compared to CE (n = 6). This provides a discrimination capacity of 0.8 for MPS compared to the 0.6 of CE. The inclusion of these highly discriminative markers to the CSYseq panel guarantees the robustness and reliability for kinship analysis or forensic familial searching. In general, the allele calls for the Y-STRs within the CSYseq ranged from 6 to 30 repeats. This wide range of allele sizes is valuable for the various demands of the panel. Y-STRs with larger allele sizes are known to exhibit larger mutation rates, which enlarges the panel’s discriminative power [14]. On the other hand, Y-STRs with short allele sizes are easier to target than long repeat stretches, since the latter are more susceptible to degradation by environmental factors and therefore often drop out of the profile in challenging samples [60]. However, this should be confirmed through additional sequencing analysis on degraded samples, such as forensically challenging or ancient DNA samples.

#### Y-STR Mutations

Through chrY comparison analysis between biologically related males for the 202 CSYseq Y-STRs, a total of 910 Y-STR differences were observed spread over 214,859 allele transfers. In total, 759 one-step, 98 two-step and 53 multi-step differences were identified which results into a ratio of 83:11:6. Remarkably, the overall percentage of two- and multi-step mutations observed in our study was more than two-fold compared to Ballantyne *et al*. with father-son couples (96:3:1) and Claerhout *et al*. with genealogical pairs (93:5:2) [14,17]. This increase could be explained by the inclusion of 20 dinucleotide RM Y-STRs in the CSYseq panel. The Y-STRs with dinucleotide repeat motifs were observed to have significant more multistep mutations. Another explanation could be the overestimation due to hidden multiple small-step mutations within the genealogical pairs [17] or to the fact that approximately 70% of the multi-step events occurred in Y-STRs located within the palindromic chrY regions. As a result, gene conversion events between palindrome arms can cause a higher occurrence of multi-step mutations [18,61]. Interestingly, even three six-step and one seven-step mutations were observed in MC Y-STRs located in the chrY palindromes. The latter explanation can be confirmed since the mutation distribution ratio when only considering Y-STR differences in single-copy loci (90:7:3) was more in line with those previously observed [14,17].

#### Y-STR mutation rates

The overall average CSYseq mutation rate of 4.57×10^−3^ mpg was in accordance with the average mutation rate for Y-STRs [14,17]. For 65 Y-STR loci, no differences were observed, even though 83% did show population variability. Literature also described 28 of them as non-mutating, while 23 were slowly mutating with an average of 1.7×10^−3^ mpg [14]. For the 136 mutating Y-STRs within our CSYseq, the calculated mutation rates for 101 Y-markers could be compared to literature, where only five of them showed a significant difference. These differences could again be explained by hidden mutation events or the rather low number of meioses analyzed for these Y-STRs due to a low depth of coverage of the amplicons. For 47 Y-STRs, only a descriptive statistical comparison could be performed based on the calibrated mutation rates by Willems *et al*. using the MUTEA (Measuring mutation rates using trees and error awareness) approach [47]. As these calibrations are population-scale evolutionary Y-STR mutation rates and lack exact generation chrY comparison, the reference mutation rate for 40 Y-STR loci fall outside our calculated 95% CI. Balanovsky (2017) already described that genealogical mutation rates are up to three times faster when compared to evolutionary mutation rates [5]. Therefore, in order to confirm these calculated mutation rates, including chrY mutation analysis with the CSYseq of more males and father-son pairs, would be beneficial. Furthermore, a significant positive correlation between the discrimination capacity and the mutation rate was observed, emphasizing the importance of keeping the CSYseq panel as diverse as possible, whereby the variability of a Y-marker is tied to its mutability rate. In accordance to the observations within literature, a positive correlation was noticed between the estimated individual mutation rate and the number of repeats as a molecular factor influencing mutability [14,17]. Additionally, through detailed repeat motif mutation analysis, it became possible to observe a higher variability in the longest repeat motifs within complex and compound Y-STRs. Also, a detailed analysis between genealogical pairs revealed three multiple and two parallel mutations which would have remained hidden through conventional CE-PCR fragment analysis. The concealment of these type of mutations can have a high impact on false tMRCA estimations for kinship research [17,30].

#### Male individualization

Using our extensive CSYseq panel, a distinction between all non-related and related males in this study could be made providing a unique Y-haplotype for every sample. Moreover, male relatives were discriminated by on average 13 Y-STR mutations, which indicates that the CSYseq panel succeeds in distinguishing related males separated by at least nine generations. This also implies that the threshold of the number of mutations to verify a biological kinship should be revised. Whereas a maximum of 10 mutations (for 40 generations) was allowed with CE using 46 Y-STRs [17], a maximum of 28 Y-STR changes on 202 Y-STR loci within the CSYseq should be allowed based on the individual CSYseq mutation rates. The highest number of Y-STR differences observed within a genealogical couple was 22 for two couples. The corresponding CE results identified nine and four Y-STR mutations for 46 Y-STRs within these genealogical pairs. Another interesting point concerns two genealogical pairs where no distinction was possible by means of CE on 46 Y-STRs, but, with our CSYseq panel discrimination turned out to be successful through the observation of 6 and 16 Y-STR differences. These mutated Y-STR loci showed a high average mutation rate of respectively 1.79×10^−2^ and 1.77×10^−2^ mpg and respectively 67 and 75% of them were observed to be RM Y-STRs. This underlines the large discriminating power that the CSYseq yields compared to CE, as well as the importance of keeping the panel as extensive as possible with the inclusion of RM Y-STRs, resulting in a unique Y-haplotype for every individual. Based on the average mutation rate of the 136 mutating Y-STRs (6.64×10^−3^ mpg), the panel has a mutation rate of 0.9 mutations per generation. In theory, this means that the CSYseq has the potential to distinguish 84% of the brothers (2 generations) and 98% of the cousins (4 generations) with at least one Y-STR difference. Yet, this needs to be confirmed by analyzing close paternal relatives (father-son, brothers) in future research.

### CSYseq robustness

Per sample, the CSYseq panel typed on average 12,281 Y-SNPs and 184 Y-STRs. This number was slightly dependent on the chrY concentration, but not with the initially identified degradation index (DI) (**S2C and S2H Fig**). For the five ‘challenging’ samples, with low chrY concentrations and high DI, over 10,800 Y-SNPs and between 127 and 182 Y-STRs were still well-typed. This reveals no clear influence of the initial concentration or DI on the success rate of MPS. Remarkably, despite the rather low chrY concentration for c1 and the high DI for d1, they both typed a high number of Y-SNPs (11,139 and 12,706) and Y-STRs (182 and 186). Therefore, it can be assumed that the input degradation and concentration requirement of the TruSeq Custom Amplicon Low Input kit is flexible and only the presence of DNA, and not the quantity, should be determined before library preparation. Although this statement needs to be confirmed by future studies with more challenging samples, this is in accordance to observations made by Poetsch *et al*. [62]. They concluded that only 0.00 ng/μl indicates the absence of DNA with the PowerQuant kit, which demonstrates that a concentration of >0.01 ng/μl could still provide a full Y-STR profile after multiplex PCR and CE. Forensic samples are often challenging due to the low amounts of DNA or high level of degradation. It can be concluded that the CSYseq Y-marker targeted resequencing is still successful with our lowest quantity DNA samples, making our CSYseq panel advantageous for challenging samples. Although high accuracy and sensitivity techniques such as MPS have already been observed as a solution compared to CE [63,64], additional analysis on degraded and low concentration samples is still recommended in order to have a more clear perspective on the precise CSYseq sensitivity.

### CSYseq applications

As the CSYseq panel exclusively targets SNPs and STRs positioned on the Y-chromosome, the output data will be valuable for a wide range of genetic-genealogical applications in interdisciplinary research as it provides valuable paternal and biogeographical background information: family history, population genetics, evolutionary biology, forensic science and even medical diagnostics.

In-depth chrY genotyping helps to unravel family history by providing more detail in patrilineal relatedness between relatives. Genealogists could make their expanded chrY-profile public or available in a database hoping to find an unexpected patrilineal relation. For population genetics, the link between a surname and patrilineage provides the opportunity to detect signals of past or recent population stratification and migrations which are still undetectable within genomic analysis of the limited number of markers [65]. Additionally, the fixed set of Y-markers sequenced with the CSYseq could avoid problems with Y-SNP nomenclature and dataset differences as is currently observed in different population studies. For molecular biology, this panel will contribute to the knowledge concerning the Y-STRs mutation rate and the molecular mechanism of mutations, together with the general understanding of Y-STR evolution in the human genome. For evolutionary biology, the CSYseq enables deeper or equal subhaplogroup determination than CE in 98% of our samples, making Y-SNP haplogroup identification through the CSYseq panel definitely more convenient. Additionally, typing a large number of Y-STRs allows to study haplogroup phylogenetics in more detail and provide valuable information for tMRCA estimations and evolutionary dating to reconstruct phylogenetic trees in more detail. Furthermore, increasing Y-STR diversity by including sequence variation in the repeat region or in the flanking regions is beneficial for molecular, evolutionary and population genetic studies as this will increase their dataset resolution.

Y-chromosome knowledge gained with the CSYseq panel also provides applications in medical diagnostics concerning male infertility which affects one in five infertile couples and one in 20 men [66]. ChrY is essential for male fertility due to the presence of several spermatogenesis-related genes. High resolution mapping of hundreds of Y-SNPs and Y-STRs will allow to identify deletions or chromosome abnormalities, helping to identify the molecular mechanisms underlying male infertility. Next to infertility, studies have shown evidence that genetic variation within the NRY could also play a part in determining cardiovascular risks as well as immune and inflammatory responses in men [20]. Correlations between the subhaplogroup and an increased disease risk were already established. Haplogroup ‘I’ shows a correlation with coronary artery disease, while haplogroup ‘N’ with infertility [20,66]. Genotyping Y-SNPs could therefore serve as a prevention analysis to identify men with an increased risk. The availability of a molecular tool to type hundreds of Y-SNPs will therefore provide the opportunity to utilize the power of phylogenetic analysis which is currently not widely used in medical genetics to explore the potential chrY contribution to complex polygenetic traits.

For forensic science, this panel can resolve some complex paternity kinship questions or provide assistance with the identification of an unknown perpetrator [2,17]. The CSYseq panel enables to find both distant paternal relatives through approximately 81 slow mutating Y-STRs (<10^−3^ mpg) and distinguish closely related individuals through 26 RM Y-STRs (≥10^−2^ mpg), which significantly increases the level of discrimination useful in forensic human identification processes [7]. This combination of Y-STRs is especially useful for familial searching, where the donor of an unknown trace has to be identified by searching for a male relative in a chrY database or through a large-scale voluntary DNA mass-screening [2,67]. Additionally, around 32 private Y-SNPs are typed which originated more recently, which makes it possible to link them to a specific population or even a single family [6]. If such a private SNP is found in a trace of the perpetrator, the number of suspects can be reduced significantly to that specific population or family. Thanks to the number of forensically interesting Y-chromosomal markers included in the CSYseq panel, a wide range of applications in the forensic field can be reached with this unique MPS panel. Through extensive Y-STR profiling, the CSYseq facilitates paternal kinship testing, disaster victim identification, cold case investigation and missing person identification [2].

MPS should become state-of-the-art in the near future to overcome the limitations of the traditionally used CE fragment analysis. CE analysis of about 15 Y-SNPs (one multiplex) and 46 Y-STRs (four multiplexes) takes multiple assays and approximately one day depending on the purification of the SNaPshot-PCR products. This does not provide detailed sequencing or subhaplogroup information as typically more than one Y-SNP multiplex has to be tested for deep subhaplogrouping. However, to implement MPS in routine DNA analysis, there are still some issues that need to be addressed concerning the nomenclature, minimum number of reads, sequencing errors, MPS strategy, data storage, minimal available MPS data and software adjustments towards new allele data [68]. To facilitate MPS in genetic research, there is also a need for specialized, expensive equipment and the improvement of the practical MPS work, as sometimes intensive laboratory effort is needed for library preparation and sequencing [69]. But, on the other hand, if this number of output needs to be obtained through CE-PCR, a much more intensive laboratory effort is needed. Furthermore, we are convinced that the low cost of less than €100 per sample (for the panel, library preparation and MiSeq run) of the CSYseq would help to apply this panel in population genetic studies worldwide.

In the end, with this study, we were able to successfully design the first extensive chrY sequencing kit. We tested the performance of the panel on samples with different concentrations and degradation levels. With the CSYseq, we offer a starting point for further investigation. To implement the kit in forensic chrY analysis, it will be necessary to further validate its repeatability and reproducibility in addition to determine its sensitivity in DNA mixture samples. Moreover, the inclusion of father-son pairs will be highly interesting to assess the discrimination power of the CSYseq panel in closely related males. This will provide additional mutability rate information for complex, compound, RM and MC Y-STRs.

## Conclusion

In this study, we developed the ‘CSYseq’ which is the first extensive Y-chromosome sequencing panel targeting 15,813 Y-markers with an easy interpretation in a single assay. A total of 9,014 Y-SNPs provide phylogenetic evolutionary information covering all main Y-haplogroups and 1,443 unique Y-subhaplogroups, which provides worldwide biogeographical background information. Additionally, a total of 202 Y-STRs are well-targeted using our CSYseq. For the search for distant family, the panel includes 81 slow mutating Y-STRs and 25 Y-STRs with low discrimination capacity (<0.1). For the discrimination of close kinships, the panel includes 46 multi-copy Y-STR loci, 14 complex or compound Y-STRs and 26 RM Y-STRs. Due to the inclusion of Y-markers with different mutation rates and discrimination powers, the CSYseq panel is diverse and highly interesting for research on different time scales: to identify evolutionary ancestry, to find distant relatives and to distinguish closely related males. In conclusion, the CSYseq enables us to sequence many interesting Y-polymorphisms covering a sufficient number of reads, an easy interpretation and a high chrY specificity, which will be valuable for interdisciplinary genetic-genealogical research worldwide.

## Materials and methods

### Ethics statement

By means of written informed consents, permission for DNA analysis and scientific publication of the anonymized results was granted. Ethical approval has been allocated by the Ethical Commission of University Hospital Leuven (S55864, S59085).

The Y-chromosome is playing an increasingly important role in evolutionary biology and population genetics [6]. However, we currently lack a universal tool for sequencing these interesting Y-polymorphisms. Until now, genotyping mostly relied on fragment analysis for Y-STRs or a single-base extension assay for Y-SNPs, which is both based on capillary electrophoresis (CE). In order to fill this gap, we present the CSYseq, the first extensive chrY-specific targeted resequencing custom-made panel targeting both evolutionary Y-SNPs as familial Y-STRs. Therefore, the CSYseq is a unique tool to indicate both distant evolutionary lineages as close familial kinships, which provides biogeographical background information and paternal lineage identification.

### Samples and DNA extraction

A total of 130 males with their residence in Belgium or the Netherlands (the Low Countries) were selected from previous studies investigating extra-pair paternity rates, haplogroup-specific Y-STR mutation rates, parallel Y-STR evolution and chrY-surname correlation [17,27,30,44,70,71]. These samples are further subdivided into five non-related males, 61 genealogical pairs with confirmed biological kinship and four extensive deep-rooted pedigrees enclosing three or four paternally related males. A total of 65 males within the study were confirmed to be unrelated. Judicial kinships between the relatives were certified per archival data and biological kinships were confirmed through 46 Y-STRs and 183 Y-SNPs genotyped using CE as described in detail within [17]. Relatives are separated by at least nine generations with an MRCA living between 1280 and 1900. Samples were collected via buccal swabs (Whatman OmniSwab, Sigma-Aldrich, USA).

For this study, DNA samples were re-extracted with the SwabSolution Kit (Promega, Madison, WI, USA). ChrY concentrations were quantified using an adapted protocol of the PowerQuant System kit (Promega, Madison, WI, USA). For 2 μl DNA-extract, 4 μl AmpSolution Reagent was added which was compensated by reducing the volume of Amplification Grade H_2_O. Samples were quantified using the Applied Biosystems 7500 Real-Time PCR System and the HID Real-Time PCR Analysis Software v1.2 (ThermoFisher Scientific, Waltham, MA, USA). qPCR was performed under the following conditions: 2’ at 98°C followed by 39 cycles of 15” at 98°C and 35” at 62°C. Autosomal and Y-chromosomal DNA concentrations together with a degradation index (DI) per sample was obtained.

### Custom panel, library preparation and sequencing

Regions of interest to target several pre-selected Y-STRs and Y-SNPs were carefully chosen based on literature. Y-STRs were selected on their sequence motif, Y-chromosome location and/or (if available) previously observed mutation rates and included all 46 Y-STRs of our in-house YForGen kit [14,17,47,59]. Y-SNPs were selected based on the Minimal reference Y-tree (www.phylotree.org/Y/tree/index.htm), containing 759 branch-defining Y-SNPs which targets 458 subhaplogroups distributed over the entire Y-SNP phylogenetic tree [10]. This resulted into a list of 865 defined chrY regions (cumulative 39,126 bp) containing 251 Y-STRs and 772 phylogenetic Y-SNPs. Selected regions ranged from 1 bp for Y-SNPs up to 950 bp for Y-STRs or a combination of Y-STRs and Y-SNPs. Primer pairs were designed using DesignStudio (Illumina, San Diego, CA, USA) with regard to the TruSeq Custom Amplicon Low Input kit with an amplicon length of 250 bp and avoiding SNPs in the primer positions (1000 Genomes as variant source). A final panel was obtained of 857 amplicons with their target length between 225 bp and 897 bp (average of 248 bp) encompassing 209,248 bp chrY sequence. Regions longer than 250 bp were covered by multiple amplicons with a combination of forward and reverse targets. In total, 228 Y-STRs and 757 Y-SNPs of the polymorphisms of interest could be genotyped with this panel.

The library preparation protocol was performed according to the guidelines of Illumina. With the TruSeq Custom Amplicon Low Input kit (Illumina, San Diego, CA, USA) a DNA input of 10 ng is recommended [49]. The custom designed primers (CAT, custom amplicon tube) were hybridized on the Veriti 96-well Thermal Cycler (ThermoFisher Scientific, Waltham, MA, USA). Unbound oligos were removed by magnetic sample purification beads (SPB) using four wash steps. PCR for primer extension and ligation was followed by library amplification while adding dual-index i7 and i5 adapters (TruSeq Custom Amplicon Index Kit, Illumina). To achieve the desired library yield and specificity, the optimal PCR cycle number for our oligo pool was 27 cycles (701–999 amplicons) according to the TruSeq kit guidelines. Libraries were again purified with SPB and washed three times to clean up other reaction components. Library quality was checked by DNA electrophoresis using the DNA 1000 kit (range 0.5–50 ng/μl) on the 2100 BioAnalyzer Instrument (Agilent, Santa Clara, CA, USA). An additional purification step was included for libraries with high primer or adapter dimers. In this step, purification beads were included in a ratio of 0.8:1 to filter out the shorter fragments (<200 bp). Libraries of approximately 370 bp and a sufficiently large peak height were normalized by qPCR DNA quantification using the KAPA SYBR Library Quantification kit for Illumina Platforms (KAPA Biosystems, Wilmington, MA, USA). P5 and P7 primers (Integrated DNA Technologies IDT, Leuven, Belgium) were diluted to 10 μM, libraries were diluted to a 1:10.000 ratio and analyzed in duplo with the Applied Biosystems 7500 Real-Time PCR System and the HID Real-Time PCR Analysis Software v1.2 (ThermoFisher Scientific, Waltham, MA, USA). A relative concentration of each sample was calculated using the comparative Ct method. For each sample, the highest Ct-value (the calibrator sample) was deducted from their Ct-value resulting in ΔCt. To determine the normalized volumes of each sample, all samples were divided by 2^-ΔCt^ and the calibrator volume was set at 10 μl to pool the libraries. The normalized pool of libraries was paired-end sequenced (2 × 300 bp), using the Miseq System and the MiSeq Reagent Kit v3 (Illumina, San Diego, CA, USA) according to the manufacturer’s protocol. In total 132 libraries were sequenced, including one female sample and one male sample twice as an internal control. Within the female sample, Y-markers can only be called by our CSYseq.analYser for Y-STRs or by Yleaf for Y-SNPs when the entire targeted region including primer positions is translocated. Sequencing and sample de-multiplexing using index-barcodes was done by Genomics Core (UZ Leuven, Belgium). The theoretical specification of 44–50 million reads per run (MiSeq Reagent Kit v3, Illumina) could be reached with 30 pM and a PhiX of 5–10%.

### Data analysis

Sample quality control was executed using FastQC v0.11.8 software (Babraham Institute, Cambridge, UK) in which the per base sequencing quality was checked. Primers were trimmed using Galaxy tools and chrY alignment was done with SAMtools (SAM file) against the GRCh37/hg19 reference genome and visualized using the Integrative Genomics Viewer 2.8.0 (IGV, BAM/BAI file) [72]. Paired-end read alignment percentage per chromosome was calculated and compared to investigate primer homology.

#### Y-SNPs and Y-subhaplogroups

Y-SNPs were analyzed using Yleaf 1.0 software [42] and an in-house Yleaf script written in MATLAB (**S6 Table**). A positions file containing 307,583 Y-SNPs adapted from the hg19 and hg38 raw data from ISOGG YBrowse database version 2019–2020 (ybrowse.org/gb2/gbrowse/chrY) was in-house developed for haplogrouping. The positions file comprises Y-SNPs along with their haplogroup, hg19 position and mutation information. We also included reported Y-SNPs that are located outside the CSYseq panel amplicon regions to call Y-SNPs sequenced by primer homology. All Y-SNPs located in the primer regions of the Illumina amplicons, having an equal or unknown mutation (e.g. T→N) and being indel or poly-allelic were excluded from the positions file as a wrong haplogroup determination could occur. A threshold of minimum 10 reads, a quality of 20 and a minimum of 90% for base result acceptance was set in Yleaf. For all samples, the subhaplogroup determined by Yleaf was compared to the previous identified subhaplogroup by SNaPshot-CE. The haplogroup coverage alongside the number of Y-SNPs targeted by the CSYseq were compared to the Minimal reference Y-tree (www.phylotree.org/Y/tree/index.htm) [10].

#### Y-STRs and Y-haplotypes

Y-STR analysis was performed using FDSTools 1.2.0 [73] and an in-house configuration file created based on our targeted amplicon chrY positions list. This configuration file contains the 5’ and 3’ anchor sequences (15 bp), the Y-STR repeat structure, motif length and prefix and suffix flanking regions. All variable repeat motifs together with possible interruptions within or between the repeat motifs were also included. These parameters were determined using the reference sequences from UCSC Genome Browser (genome.ucsc.edu) and YBrowse (ybrowse.org/gb2/gbrowse/chrY). The Y-STR repeat sequence was determined by comparison with literature and according to the rules of Kayser *et al*. where at least three homogeneous repetitions have to be included in the repeat sequence [14,47,59]. Using SAMtools (Sequence Alignment/Map), the amplicon positions provided by Illumina were checked [74]. Additional repeating sequences were searched using Tandem Repeat Finder (TRF) [43] within all high quality (>40 MAPQ) reads aligning to chrY in order to identify additional Y-STRs present in the CSYseq panel. The repeat structure of the yet unknown Y-STR loci was defined using the STRNaming tool [75]. All FASTQ files were analyzed using the standard FDSTools pipeline and an in-house developed AutoExecuter using Python in order to easily analyze multiple FASTQ files at once (**S7 Table**). For both single-end reads, three output files were created: a CSV-file containing all raw data, a HTML-file which displays a user-definable visual overview of all Y-STRs and a text-file. The text file includes for every unique sequence of each locus the name, the sequence and the number of reads separately for the forward and reverse strand. The CSV-file was further used to uncover exact Y-STR sequences of the repeat and flanking regions (prefix and suffix), together with the genuine allele call.

As the CSV-file provides all unique sequence reads for each Y-STR locus, a lot of data still needs manual analysis by the user to uncover the genuine allele call. Therefore, an analysis file, called the ‘CSYseq.analYzer’, was created in Excel using Visual Basic Assistant and Excel Macros. The CSYseq.analYzer is publicly available and included as a supplementary Excel file. The CSYseq.analYser filters out stutter sequences among the raw data and selects the most probable allele call with the highest number of reads. This ‘CSYseq.analYzer’ excludes sequencing errors or reads with a low depth of coverage by providing a ‘no data’-label and it simultaneously corrects wrong Y-STR data output created due to for instance primer homology. To establish this, the sequence covered by the highest number of reads is divided in ‘prefix’, ‘repeat’ and ‘suffix’. If, due to homology or sequencing errors, the flanking regions are too long or too short to be the actual amplicon, this was repeated for the sequence with the second, third and fourth highest number of reads. If the fourth one was again incorrect, the output for this Y-STR was labeled as ‘no data’. This means that only the sequences covered by the four highest number of reads were checked. For the multi-copy Y-STRs this was multiplied by the number of copies. Y-STR allele calls and given sequences by FDSTools were double checked using IGV. Through the basic local alignment search tool (BLAST) on IGV, primer homology intra or inter chromosomes could be confirmed. The CSYseq.analYzer contains three visible worksheet tabs: two ‘input’ tabs for the single-end CSV output of FDSTools and one ‘output’ tab providing all the results. Besides, four worksheet tabs are hidden, since they are not needed for the user to manipulate when sequencing with the CSYseq kit. These hidden worksheet tabs concern two tabs which contain our CONFIG file used for FDSTools and two tabs include all the calculations needed to create the output. As a result, the ‘CSYseq.analYzer’ provides a simple and convenient overview table with the most feasible sequence read information for each Y-STR locus (single-end and paired-end). The output enlists the Y-STR allele call and motif, the corresponding Y-STR prefix and suffix, and a sequence variance column including sequence variations compared to GRCh37/hg19 from UCSC Genome Browser.

Three internal control steps were included in order to obtain the most reliable genotyping results. First, a paired-end consensus was made based on the results from the single-end reads (R1 and R2). When R1 and R2 resulted in a different allele call, the read encountering the Y-STR close to its starting anchor sequence was selected to be the most reliable read as sequencing errors increase per cycle and thus per base. For equal R1 and R2 Y-STR positions, the one encountering the highest depth of coverage was selected. Second, the genotype concordance for 21 Y-STR loci of the CSYseq was compared to previously obtained PCR-CE results from our in-house YForGen kit and commercial Y-kits [44]. This is also an extra internal control step against a possible sample-library switch. Based on the conformity between the CE results and MPS reads, a consideration about the stutter frequency could be made for some multi-copy Y-markers. Third, the male sample sequenced twice was checked for matching MPS output. For all well-typed Y-STRs, non-related males were analyzed in order to gain information about the discrimination capacity, allele call frequencies and allele ranges using GenAlEx 6.51b2 [76]. By chrY comparison within the genealogical pairs, average and individual Y-STR mutation rates with their 95% confidence intervals were calculated based on the frequentist approach through direct counting of the number of observed Y-STR differences divided by the total number of meioses [77]. Results were compared to previously defined mutability rates from literature using the Chi-square test [14,48,78,79]. Previously identified influencing molecular factors (repeat size, repeat motif length and repeat type) could be further investigated.

CE Y-chromosomal data in this study has been submitted previously to the open access Y-STR Haplotype Reference Database (YHRD, https://yhrd.org) available under accession numbers YA003651-53, YA003739-42 and YA004300-01.

## Supporting information

S1 Fig A heat map of the target chromosome distribution. The number of aligned single-end reads per library (rows) sorted on chrY alignment percentage.
(TIFF)

S2 Fig CSYseq robustness. **A.** Schematic overview of the Figure panels. **B.** DNA quantification by PowerQuant qPCR before library preparation. Red lines: thresholds 2.5 ng/μl and DI of 2; d1: highest DI; c1: lowest concentration. **C.** Library quality using the BioAnalyzer. **D.** KAPA qPCR library Ct values. **E.** FASTQ reads per library. **F.** (left) FASTQC read position when quality Phred scores of the lower quartile goes below 20. (right) FASTQC outputs of d1 and c1. **G.** Typed Y-SNPs using Yleaf. **H.** Typed Y-STRs using FDSTools.
(TIFF)

S3 Fig The Y-SNP haplogroup distribution. Distribution in Belgium and the Netherlands (Low Countries) and the typed Y-SNP haplogroup distribution of the CSYseq subdivided into ancestral and derived typed Y-SNPs.
(TIFF)

S1 Table A complete phylogenetic tree including all CSYseq typed Y-subhaplogroups. (XLSM)

S2 Table Detailed information concerning the 28 Y-STR loci excluded from the CSYseq panel. (XLSM)

S3 Table HGVS nomenclature for the CSYseq Y-STRs. (XLSM)

S4 Table Multi-copy dinucleotide Y-STRs. The interpretation of the different stutter and allele peak combinations for the multi-copy dinucleotide Y-STR YCAII-ab.
(XLSM)

S5 Table Double repeat sequence variability for the compound and complex Y-STRs included in the CSYseq. (XLSM)

S6 Table Yleaf analyszing script. A script developed in MATLAB in order to analyze FASTQ files using Yleaf 1.0. For each Y-SNP, a threshold of minimum 10 reads, a quality of 20 and a minimum percentage of base result for acceptance of 90 was set.
(XLSM)

S7 Table AutoExecuter. The in-house developed AutoExecuter written in Python in order to easily analyze multiple FASTQ files at once with the standard FDSTools pipeline.
(XLSM)
