# Supplementary figures and images for: CSYseq: The first Y-chromosome sequencing tool typing a large number of Y-SNPs and Y-STRs to unravel worldwide human population genetics
Source: PLoS Genet. 2021 Sep 7;17(9):e1009758. doi: 10.1371/journal.pgen.1009758 (PMC8423258; doi:10.1371/journal.pgen.1009758)

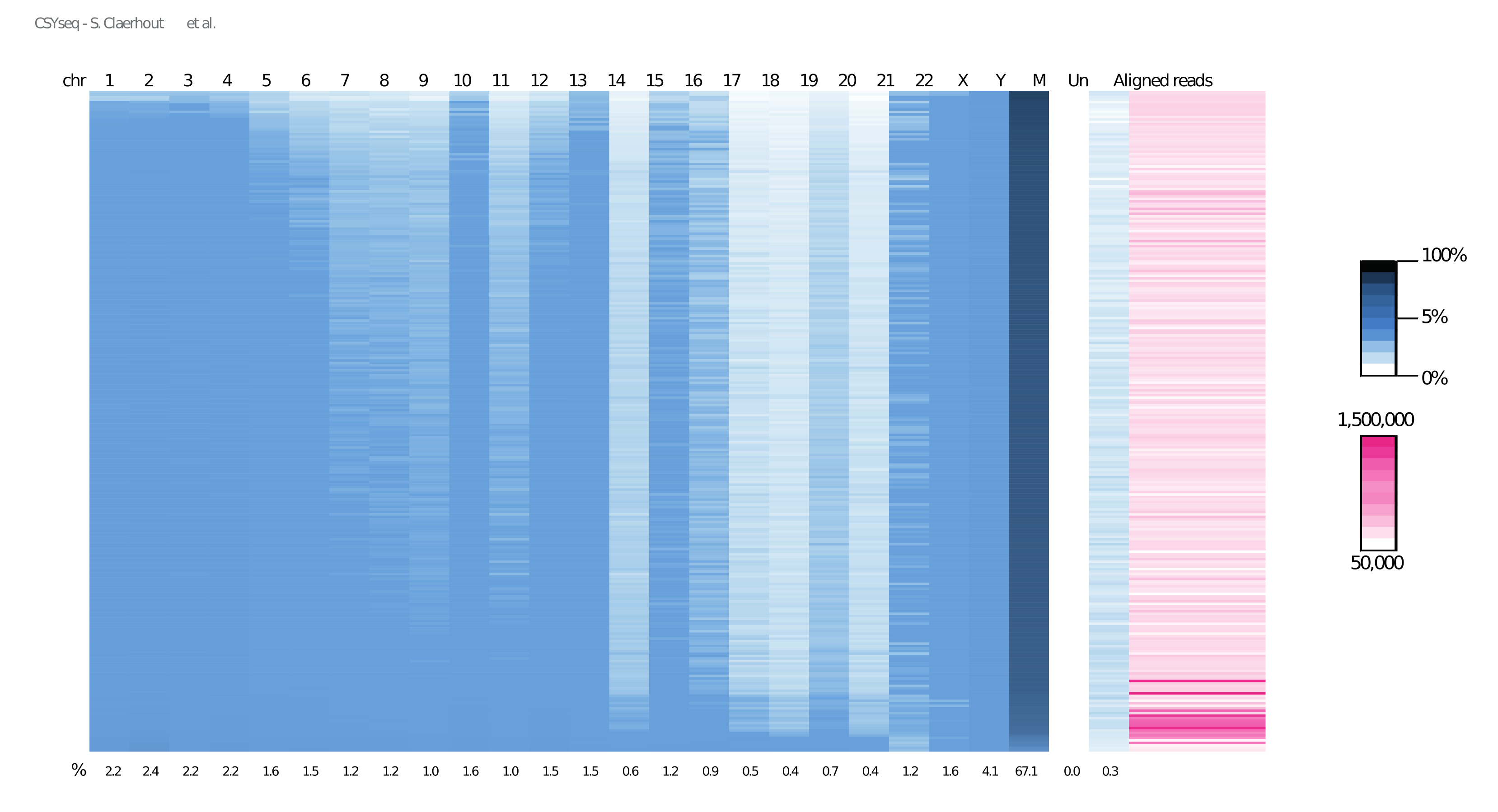

Supplement: S1 Fig — The number of aligned single-end reads per library (rows) sorted on chrY alignment percentage. (TIFF) [file pgen.1009758.s001.tiff]

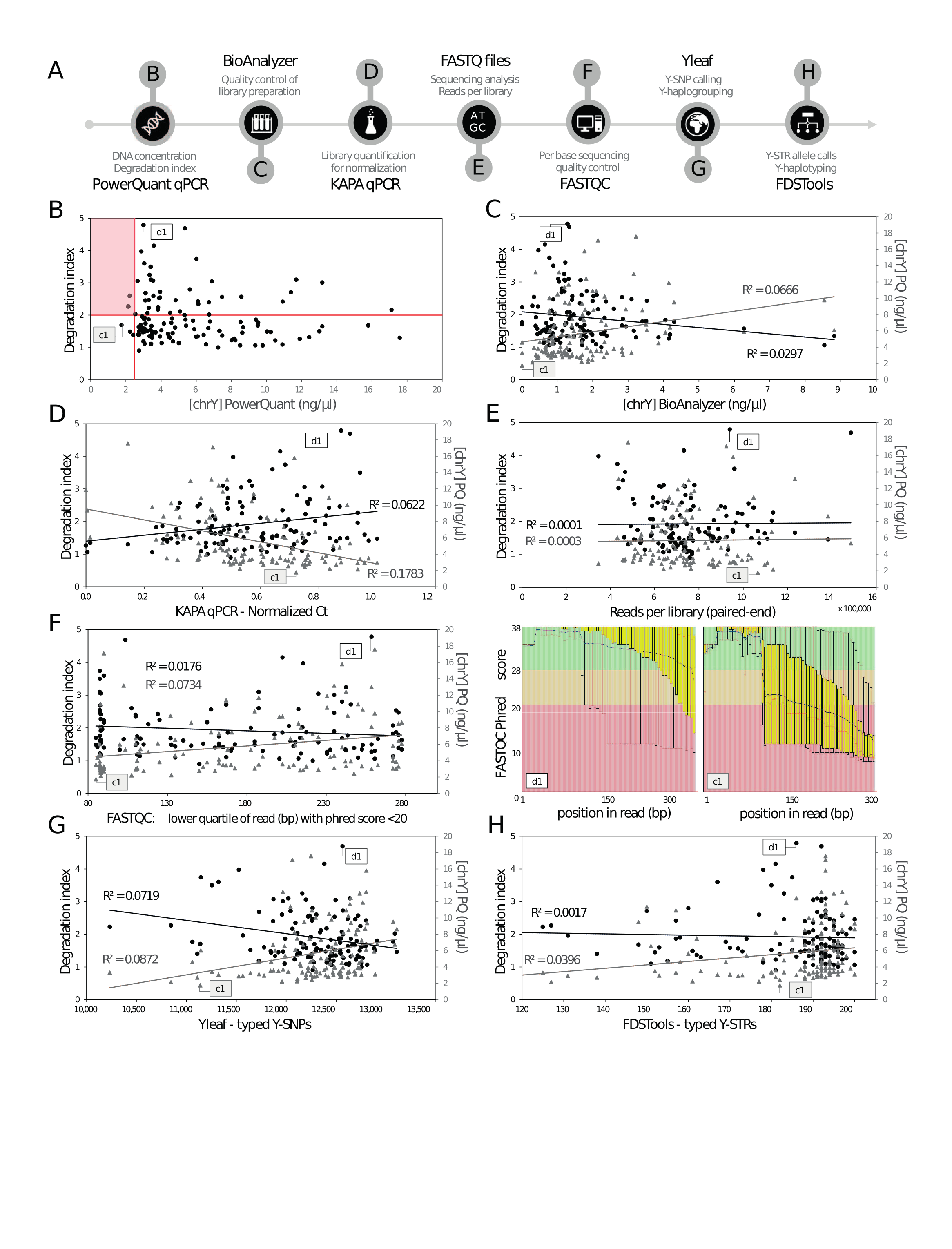

Supplement: S2 Fig — A. Schematic overview of the Figure panels. B. DNA quantification by PowerQuant qPCR before library preparation. Red lines: thresholds 2.5 ng/μl and DI of 2; d1: highest DI; c1: lowest concentration. C. Library quality using the BioAnalyzer. D. KAPA qPCR library Ct values. E. FASTQ reads per library. F. (left) FASTQC read position when quality Phred scores of the lower quartile goes below 20. (right) FASTQC outputs of d1 and c1. G. Typed Y-SNPs using Yleaf. H. Typed Y-STRs using FDSTools. (TIFF) [file pgen.1009758.s002.tiff]

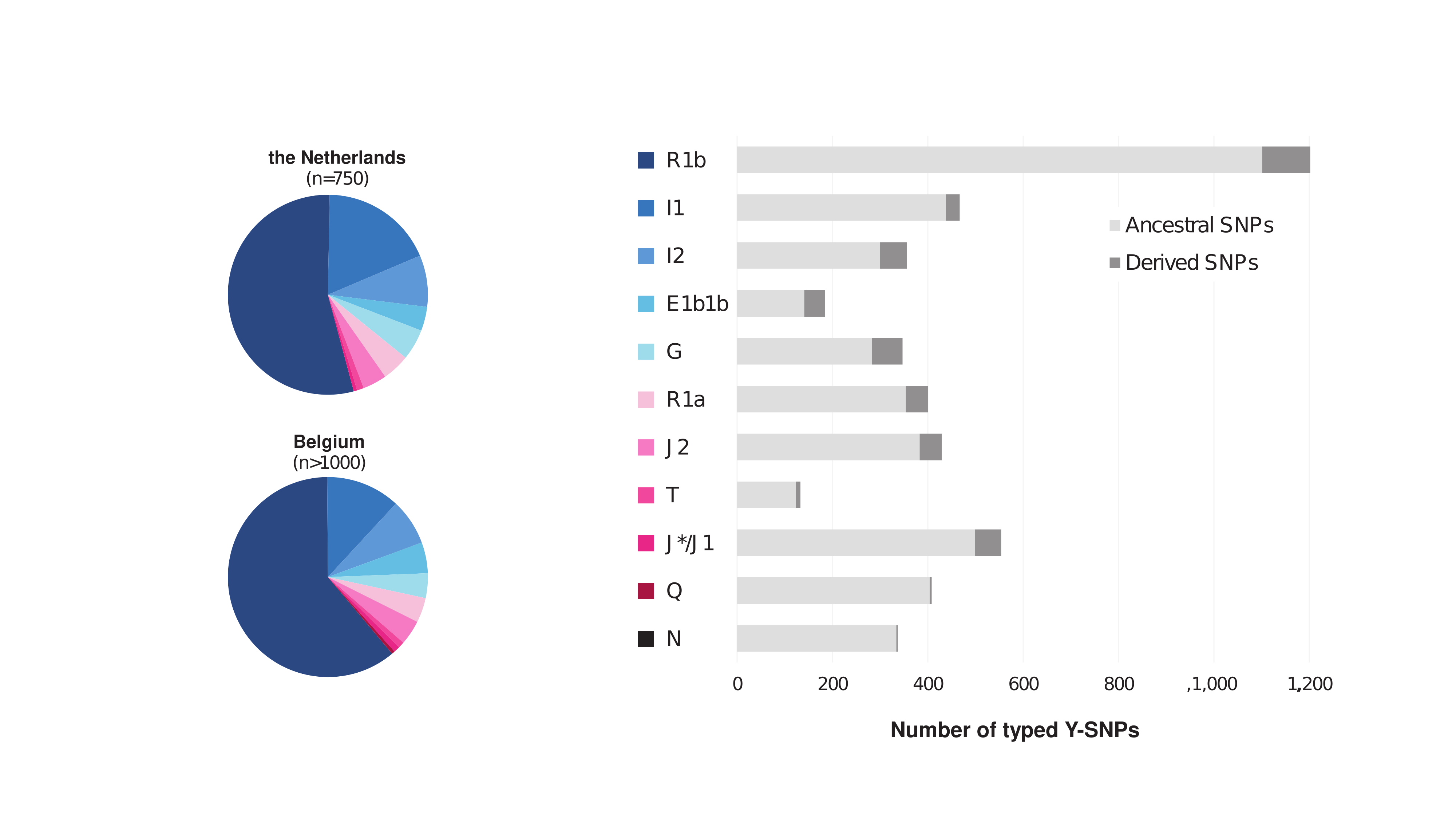

Supplement: S3 Fig — Distribution in Belgium and the Netherlands (Low Countries) and the typed Y-SNP haplogroup distribution of the CSYseq subdivided into ancestral and derived typed Y-SNPs. (TIFF) [file pgen.1009758.s003.tiff]
